# Paralog-Specific Functions of *RPL7A* and *RPL7B* Mediated by Ribosomal Protein or snoRNA Dosage in *Saccharomyces cerevisiae*

**DOI:** 10.1534/g3.116.035931

**Published:** 2016-12-19

**Authors:** Ryan J. Palumbo, Gabriele Fuchs, Sheila Lutz, M. Joan Curcio

**Affiliations:** * Laboratory of Molecular Genetics, Wadsworth Center, New York State Department of Health, Albany, New York 12201; † Department of Biomedical Sciences, School of Public Health, University at Albany, New York 12201; ‡ Department of Biological Sciences, The RNA Institute, University at Albany-State University of New York, New York 12222

**Keywords:** ribosomal protein, paralog, isoform, retrotransposon, RNA localization

## Abstract

Most ribosomal proteins in *Saccharomyces cerevisiae* are encoded by two paralogs that additively produce the optimal protein level for cell growth. Nonetheless, deleting one paralog of most ribosomal protein gene pairs results in a variety of phenotypes not observed when the other paralog is deleted. To determine whether paralog-specific phenotypes associated with deleting *RPL7A* or *RPL7B* stem from distinct functions or different levels of the encoded isoforms, the coding region and introns of one paralog, including an intron-embedded snoRNA (small nucleolar RNA) gene, were exchanged with that of the other paralog. Among mutants harboring a single native or chimeric *RPL7* allele, expression from the *RPL7A* locus exceeded that from the *RPL7B* locus, and more Rpl7a was expressed from either locus than Rpl7b. Phenotypic differences in tunicamycin sensitivity, *ASH1* mRNA localization, and mobility of the Ty1 retrotransposon were strongly correlated with Rpl7 and ribosome levels, but not with the Rpl7 or snoRNA isoform expressed. Although Ty1 RNA is cotranslationally localized, depletion of Rpl7 minimally affected synthesis of Ty1 Gag protein, but strongly influenced Ty1 RNA localization. Unlike the other processes studied, Ty1 cDNA accumulation was influenced by both the level and isoform of Rpl7 or snoRNA expressed. These cellular processes had different minimal threshold values for Rpl7 and ribosome levels, but all were functional when isoforms of either paralog were expressed from the *RPL7A* locus or both *RPL7* loci. This study illustrates the broad range of phenotypes that can result from depleting ribosomes to different levels.

The *Saccharomyces cerevisiae* ribosome comprises four ribosomal RNAs and 79 ribosomal proteins (RPs) synthesized in nearly equimolar amounts. Notably, 59 RPs are encoded by paralogous gene pairs that arose from a whole genome duplication and were selectively retained (Wolfe and Shields 1997). Paralogs may persist in the genome because they encode redundant functions that balance gene dosage, become specialized for different environmental conditions, or acquire distinct functions by subfunctionalization or neofunctionalization (Innan and Kondrashov 2010; Parenteau *et al.* 2015). Most RP paralogs are redundant for a ribosome-associated function that is essential for optimal cell growth or viability (Dean *et al.* 2008; Steffen *et al.* 2012; Woolford and Baserga 2013). The mRNAs transcribed from these genes, although heterogeneous in sequence, especially in untranslated regions (UTRs) (Leer *et al.* 1985), typically encode proteins of identical, or nearly identical, length and amino acid sequence. Fitness defects resulting from deletion of one paralog can be suppressed by ectopic expression of the coding region of the other paralog from an active promoter (Abovich and Rosbash 1984; Rotenberg *et al.* 1988; Simoff *et al.* 2009). Nonetheless, deletion of one paralog or the other of an RP gene pair often results in distinct transcriptional and phenotypic profiles (Komili *et al.* 2007). A single paralog of discrete subsets of RP genes is required for bud-site selection (Ni and Snyder 2001), translational repression, and bud tip localization of the *ASH1* mRNA (Komili *et al.* 2007), actin organization (Haarer *et al.* 2007), vacuolar carboxypeptidase Y secretion (Bonangelino *et al.* 2002), propagation of the M_1_ satellite dsRNA of the L-A virus (Ohtake and Wickner 1995), protection from killer toxin (Page *et al.* 2003), telomere length control (Askree *et al.* 2004), replicative life span (Steffen *et al.* 2008, 2012), and mobility of the Ty1 retrotransposon (Dakshinamurthy *et al.* 2010; Risler *et al.* 2012). The divergent phenotypes associated with deleting one or the other paralog are not always correlated with the relative abundance of mRNA or RP produced from individual paralogs in a wild-type strain (Komili *et al.* 2007; Steffen *et al.* 2008, 2012). For example, it was argued that *RPS18B* is specifically required for *ASH1* mRNA localization because deletion of *RPS18B* but not *RPS18A* abolished localization despite equivalent levels of epitope-tagged Rps18a and Rps18b in a wild-type strain (Komili *et al.* 2007). Collectively, these observations led to the “ribosome code” hypothesis, which posits that the divergent RP isoforms are incorporated in different combinations into heterogeneous ribosomes that selectively translate specific subsets of mRNAs (Mauro and Edelman 2002, 2007; Komili *et al.* 2007). The specialized functions of heterogeneous ribosomes may be further diversified by selective post-translational modification of RPs and ribosomal RNAs (Mauro and Edelman 2002, 2007; Gilbert 2011; Xue and Barna 2012).

Discordance between the phenotypic consequences of deleting each paralog, and the relative level of mRNA or RP produced from each paralog in a wild-type strain could result from compensatory changes in the RP level produced from one paralog when the other is deleted. In one study, the longer replicative life span of an *rpl20b∆* mutant was correlated with a lower level of 60S ribosomal subunits compared to an *rpl20a∆* mutant, even though *RPL20A* and *RPL20B* mRNA levels are equivalent in a wild-type strain. Thus, a disparity in Rpl20 levels in *rpl20a∆* *vs.* *rpl20b∆* mutants could explain the paralog-specific role of *RPL20B* in replicative life span (Steffen *et al.* 2008). Given that RP paralog expression levels are often altered by deletion of the other paralog (Eng and Warner 1991; Presutti *et al.* 1991; Paulovich *et al.* 1993; Li *et al.* 1995; Fewell and Woolford 1999; Badis *et al.* 2004), the question of whether paralog-specific phenotypes result from RP isoforms with disparate functions, differences in the level or activity of the RP produced from each paralog, or a combination of these mechanisms has not yet been rigorously tested.

To address this question, we have examined phenotypes associated with expressing *RPL7A* or *RPL7B* at different levels. Rpl7 isoforms participate in the earliest steps of 60S precursor rRNA processing (Jakovljevic *et al.* 2012), and bind to 25S and 5S rRNAs in the mature 60S ribosomal subunit (Ben-Shem *et al.* 2011). Five of the 244 amino acid residues in Rpl7a and Rpl7b are divergent, with four amino acid substitutions in Rpl7b relative to Rpl7a (A2S, A3T, S16T, and V26I) being in the conserved N-terminal domain that is predicted to be on the surface of the ribosome (Lin 1991; Tsai *et al.* 2012). The possibility that Rpl7a and Rpl7b have unique functions as components of heterogeneous ribosomes was suggested by the involvement of different ribosome biogenesis factors in assembly of ribosomes containing Rpl7a *vs.* Rpl7b (Komili *et al.* 2007). In addition, the *RPL7A* and *RPL7B* genes are unique among RP paralogs in that each contains a paralogous C/D box snoRNA gene, *snR39* or *snR59*, that is encoded in the second intron (Figure 1A), and likely expressed from the excised intron (Vincenti *et al.* 2007). While divergent in sequence, snR39 and snR59 function redundantly as guide RNAs for 2′-O-methylation of residue A807 in the large subunit rRNA (Piekna-Przybylska *et al.* 2007). Deletion of one or both of the introns harboring these snoRNA genes has no effect on cell viability (Parenteau *et al.* 2011). Several paralog-specific functions have been assigned to *RPL7A* and *RPL7B*; however, they are asymmetrically expressed in a wild-type strain (Ghaemmaghami *et al.* 2003; Komili *et al.* 2007), and the *rpl7a∆* mutant has a diminished level of 60S ribosomal subunits, whereas *rpl7b∆* has a wild-type polysome profile (Jakovljevic *et al.* 2012). Thus, it remains to be determined whether Rpl7a or Rpl7b has unique functions irrespective of its expression level.

**Figure 1:**
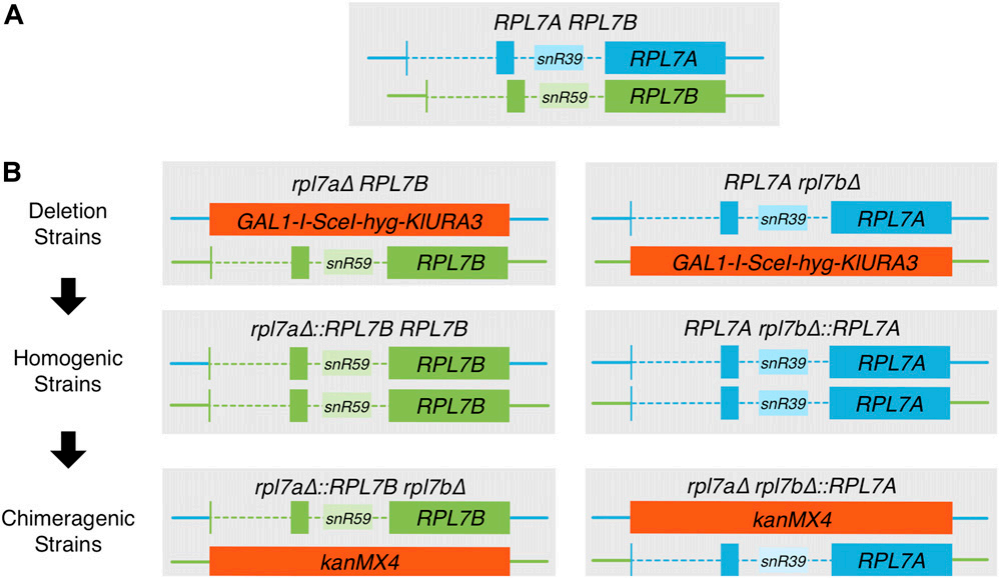
Schematic of the *RPL7A* and *RPL7B* loci used in this study. (A) *RPL7A* and *RPL7B* coding regions are interrupted by two introns. Encoded in the second intron is a snoRNA paralog, *snR39* or *snR59*. (B) Construction of chimeragenic *rpl7a∆*::*RPL7B rpl7b∆* (left column), and *rpl7a∆ RPL7B∆*::*RPL7A* (right column) mutants from the wild-type strain in three sequential steps. First, the *RPL7A* or *RPL7B* CRI was replaced with the *GAL1-I-SceI-hygB-KlURA3 delitto perfetto* cassette. Second, replacement of the *delitto perfetto* cassette with the *RPL7B* or *RPL7A* CRI, respectively, generated homogenic strains harboring the same CRI at both *RPL7* loci. Third, the CRI of the *RPL7B* or *RPL7A* locus in homogenic strains was replaced with the *kanMX4* marker to generate chimeragenic strains. Turquoise represents sequences derived from the *RPL7A* gene, and green represents sequences derived from the *RPL7B* gene. Solid, colored lines: noncoding locus DNA flanking the CRI; dashed, colored lines: introns; solid, dark-colored boxes: exons; solid, light-colored boxes: intron-encoded snoRNA genes.

Because selective pressure to suppress retrotransposon activity in the genome can drive neofunctionalization of gene paralogs involved in retrotransposon control (Münk *et al.* 2012), we examined the role of *RPL7A* and *RPL7B* in regulating Ty1, a long terminal repeat (LTR)-retrotransposon present in ∼30 copies in haploid *S. cerevisiae* strains. Ty1 elements contain two overlapping ORFs: *GAG*, which encodes the capsid protein, and *POL*, which is expressed by translational frameshifting, and encodes enzymatic mobility proteins. Retromobility involves localized translation of Ty1 RNA in cytoplasmic foci known as retrosomes, assembly of Ty1 RNA and proteins into virus-like particles (VLPs), reverse transcription of Ty1 RNA within VLPs, transport of cDNA to the nucleus, and introduction of the cDNA into the host genome by nonhomologous integration or homologous recombination [reviewed in Curcio *et al.* (2015)]. Deletion of *RPL7A* results in lower levels of Ty1 retrosomes, cDNA, and retromobility (Risler *et al.* 2012; Doh *et al.* 2014).

In this study, the coding region and introns (CRI) of *RPL7A* and *RPL7B* were exchanged to express Rpl7 and snoRNA isoforms at different levels. Expression of either *RPL7* CRI from the *RPL7A* locus resulted in faster cell growth and higher levels of Rpl7 and 60S ribosomal subunits than expression from the *RPL7B* locus. Moreover, the *RPL7A* CRI is expressed more highly from either locus than the *RPL7B* CRI. Divergent growth rates, Rpl7 levels and 60S/40S ribosomal subunit ratios in strains expressing a single native or chimeric allele of *RPL7* correlated strongly with differences in sensitivity to tunicamycin, efficiency of *ASH1* mRNA and Ty1 retrotransposon RNA localization, and rate of Ty1 retromobility. Another phenotype, Ty1 cDNA accumulation, correlated with both the level and isoforms of Rpl7 and snoRNA expressed. These cellular processes required different minimal levels of Rpl7 or the *RPL7*-encoded snoRNA, but all phenotypic defects resulting from either *RPL7* CRI being expressed from the *RPL7B* locus, or from the *RPL7B* CRI being expressed from the *RPL7A* locus were suppressed by expressing the *RPL7B* CRI from both loci. Thus, Rpl7 and snoRNA isoforms encoded by *RPL7A* and *RPL7B* have redundant, dosage-dependent functions. Overall, the findings suggest that cellular processes have different minimal threshold-values for the level of Rpl7 or snR39/59, and, consequently, 60S ribosomal subunits that are required, and that variations in 60S ribosomal subunit levels underlie the phenotypic diversity that results from deleting one *RPL7* paralog or the other.

## Materials and Methods

### Yeast strains, media, and plasmids

All synthetic complete (SC) drop-out media contained 2% glucose unless otherwise noted. The strains used in this study are derivatives of strain BY4741 (Open Biosystems), and genotypes are listed in Supplemental Material, Table S1. Strain JC3212 harbors a chromosomal Ty1*his3AI[∆1]*-3114 element (Mou *et al.* 2006). Strain JC3807 is a derivative of JC3212 harboring Ty1(*GAG:GFP*)-3566 (Scholes *et al.* 2003). Oligonucleotide primers used to amplify PCR products for gene replacement are listed in Table S2. PCR products used for gene replacement were amplified with Phusion High Fidelity Polymerase (New England Biolabs). Each gene replacement was confirmed by two independent PCR analyses.

Plasmid pGSHU, a gift from Michael Resnick, harbors the *GAL1_P_ -I-SceI-hygB-K.l.URA3* counter-selectable *delitto perfetto* cassette (Storici and Resnick 2006). A PCR-amplified *rpl7a∆*::*GAL1_P_ -I-SceI-hygB-K.l.URA3* cassette was generated with primers PJ813 and PJ814 using plasmid pGHU as a template. The cassette was introduced into strain JC3212 by one-step gene replacement, and hygromycin-resistant (Hyg^R^) Ura^+^ isolates were selected and tested for the presence of the *rpl7a∆*::*GAL1_P_ -I-SceI-hygB-K.l.URA3* allele. An *rpl7a∆*::*RPL7B* cassette, amplified by PCR using genomic DNA of an *rpl7a∆ RPL7B* strain as a template, and primers PJ757 and PJ759, was introduced into *rpl7a∆*::*GAL1_P_ -I-SceI-hygB-K.l.URA3* strains JC5896 and JC5898 by one-step gene replacement. The *RPL7B* allele was deleted from this strain using an *rpl7b∆*::*kanMX* cassette that was PCR-amplified using primers PJ463 and PJ464, and genomic DNA from an *rpl7b∆*::*kanMX* derivative of strain BY4741 as a template. A *rpl7b∆*::*GAL1_P_ -I-SceI-hygB-K.l.URA3* cassette was amplified with primers PJ842 and PJ843 using plasmid pGHU as a template, and introduced into strain JC3212 by one-step gene replacement. An *rpl7b∆*::*RPL7A* cassette, generated by PCR with primers PJ845 and PJ846 and genomic DNA from an *RPL7Arpl7b∆* strain as a template, was used to replace the *rpl7b∆*::*GAL1_P_ -I-SceI-hygB-K.l.URA3* allele in strains JC5900 and JC5901. The *RPL7A* allele was deleted from this strain using an *rpl7a∆*::*kanMX* cassette that was PCR-amplified using primers PJ459 and PJ460 and genomic DNA from the *rpl7a∆*::*kanMX* derivative of strain BY4741 as a template. An *rpl31a∆*::*GAL1_P_-I-SceI-hygB-K.l.URA3* cassette, amplified with primers PJ893 and PJ894 using plasmid pGHU as a template, was introduced into strain JC3212 by gene replacement. An *rpl31a∆*::*RPL31B* allele, generated by PCR amplification with primers PJ895 and PJ896 using genomic DNA of an *rpl31a∆ RPL31B* strain as a template, was used to replace the *rpl31a∆*::*GAL1_P_-I-SceI-hygB-K.l.URA3* allele in strains JC6130 and JC6131. The *RPL31B* allele in this strain was deleted by one-step gene replacement using a DNA fragment that was PCR-amplified with primers PJ471 and PJ472, and genomic DNA from the *rpl31b∆*::*kanMX* derivative of strain BY4741 as a template. The *tif4631∆*::*kanMX* derivative of strain JC3212 was made by gene replacement using a cassette that was PCR-amplified using primers PJ371 and PJ372, and the *tif4631∆*::*kanMX* derivative of strain BY4741 as a template. Strains harboring the *rad52*::*hisG-URA3-hisG* allele were constructed by one-step gene replacement using a DNA fragment isolated from plasmid pBDG542, as described previously (Curcio and Garfinkel 1994).

Derivatives of yeast strain JC3709 (Table S2) harboring *9xMYC*-tagged alleles of *RPL7A* and *RPL7B* were made by using PCR-amplified *RPL7A:9xMyc-K.l.TRP1* and *RPL7B:9xMyc-K.l.TRP1* cassettes for one-step gene replacement. The *RPL7A:9xMyc-K.l.TRP1* cassette was amplified using primers PJ564 and PJ565, and plasmid pYM6 (Knop *et al.* 1999) as a template. The *RPL7B:9xMyc-K.l.TRP1* cassette was amplified using primers PJ566 and PJ567, and plasmid pYM6 as a template. The corresponding untagged paralog was deleted in each strain by one-step gene replacement using a PCR-amplified *rpl7a∆*::*kanMX* or *rpl7b∆*::*kanMX* cassette, respectively. Construction of these deletion cassettes is described above.

pBJC1058, a *LEU2-CEN6* plasmid that contains the pLTR_P_:Gag_1–401_:GFP:*ADH1*_TER_ cassette, has been described previously (Doh *et al.* 2014).

Plasmid pGTy1(*synGAG*) consists of pRS416 carrying a *GAL1_P_*-driven Ty1 element that encodes functional *GAG* and *POL* ORFs, but carries silent nucleotide substitutions in the 5′ UTR and *GAG* ORF, and lacks the 3′ LTR. Plasmid pGTy1(*synGAG*) is a derivative of plasmid pBJC838 (Stamenova *et al.* 2009). The transcriptional start site of Ty1 (nucleotide 241 of Ty1-H3; Boeke *et al.* 1988) is fused to *GAL1_P_* at a *Xho*I site. In addition, the 3′ LTR and *his3AI* marker gene were deleted by removal of a DNA fragment from the internal *Bgl*II site to the end of the element (nucleotides 5564–5918 of Ty1-H3). Finally, a sequence-optimized 577 bp *Xho*I–*Hpa*I fragment has replaced the *Xho*I–*Hpa*I fragment of Ty1-H3 (nucleotides 241–817 of Ty1-H3). The sequence-optimized fragment, synthesized by GenScript, harbors multiple nucleotide changes in the Ty1 RNA 5′ UTR region to reduce predicted secondary structure elements, as well as silent mutations in *GAG* predicted by the OptimumGene algorithm (GenScript) to optimize gene expression (Figure S1). The *GAL1_P_*:Ty1(*synGAG*) fragment is carried as an *Apa*I–*Eag*I fragment on the *LEU2-CEN6* vector pRS415.

Plasmid pBJC1198 contains the *GAL1*_P_-*ASH1*-MS2L(x12)-*ADH1_TER_* cassette on the *LEU2-CEN6* vector, pRS415. The *ASH1* ORF (from the start codon to the stop codon) was amplified from BY4741 genomic DNA with primers PJ1285, which introduced a 5′ *Xho*I site, and PJ1286, which introduced a 3′ *Bam*HI site. The *Apa*I–*Xho*I fragment of plasmid pBJC838 containing the *GAL1_P_* (Stamenova *et al.* 2009), the *Xho*I–*Bam*HI fragment containing the *ASH1* ORF, and the *Bam*HI–*Bgl*II fragment of pSL-MS2-12X (Bertrand *et al.* 1998) were cloned into pRS415 (Sikorski and Hieter 1989), following digestion with *Apa*I and *Bam*HI. An *Eag1* fragment harboring *ADH1* terminator sequences (*ADH1_TER_*) was cloned into the *Eag*I site of the resulting plasmid. *ADH1_TER_* was amplified from plasmid pBJC1058 with primers PJ1169 and PJ944.

Plasmid pMS2-CP-GFP(3X), a gift from Jeffrey Gerst, expresses the MS2 coat binding protein fused to three tandem GFP moieties, and is driven by the *MET15* promoter (Haim-Vilmovsky and Gerst 2009). Basal expression of pMS2-CP-GFP(x3) in the presence of methionine was sufficient to visualize p*ASH1-MS2L*(x12) mRNA while minimizing background signal.

### Western blot analyses

Culture volumes corresponding to 2.5 OD_600_ units of cells grown to an OD_600_ of 0.6–1.0 at 20° were harvested, and protein was extracted as previously described (Yarrington *et al.* 2012). Proteins were separated on 10% SDS-Tris gels and transferred to an Immun-Blot PVDF membrane (Bio-Rad). To detect Gag, membranes were incubated with affinity-purified anti-VLP polyclonal antibodies (Conte and Curcio 2000), at a 1:35,000 dilution in 0.005% nonfat milk in phosphate-buffered saline and 0.05% Tween 20 (PBST), followed by incubation with horseradish peroxidase (HRP)-conjugated goat anti-rabbit secondary polyclonal antibody (Millipore) in 0.005% nonfat milk in PBST. Rpl7a-9xMyc and Rpl7b-9xMyc were detected with a 1:5000 dilution of monoclonal anti-c-Myc antibody (clone 9E10; Sigma), followed by incubation with HRP-conjugated sheep anti-mouse secondary polyclonal antibody (GE Healthcare UK Limited; lot # 357597). Membranes were stripped in 50 mM Tris-HCl pH 7, 2% SDS, 50 mM DTT at 70°. Membranes originally used to detect Gag were incubated in incubated in a 1:2000 dilution of anti-humL7 polyclonal antibodies (Bethyl Labs, Inc.) in PBST, and then HRP-conjugated goat anti-rabbit secondary polyclonal antibodies (Millipore) in PBST. After stripping, the membrane was incubated in a 1:2000 dilution of anti-β-Actin monoclonal antibody (Abcam) in PBST with 2.5% nonfat milk, followed by incubation with a 1:4000 dilution of HRP-conjugated sheep anti-mouse secondary polyclonal antibody (GE Healthcare UK Limited; lot # 357597) in PBST with 2.5% nonfat milk. Membranes originally used to detect Rpl7a-9xMyc and Rpl7b-9xMyc were incubated in a 1:2000 dilution of anti-α-Tubulin polyclonal antibody (Chemicon International) in PBST with 2.5% nonfat milk, followed by incubation in a 1:5000 dilution of HRP-conjugated donkey anti-rat secondary polyclonal antibody (Millipore; lot # 2050222) in PBST with 2.5% nonfat milk. All bands were visualized by incubation of membranes in SuperSignal West Pico chemiluminescent substrate (Pierce) and exposure to film. Films were scanned, and the intensity of nonsaturated bands was determined using ImageJ software. Gag and Rpl7 values were individually divided by the values for β-Actin, and corrected for differences in dilution.

### Growth assays

To determine the rate of cell doubling of each strain, cells were scraped from fresh patches on YPD agar, and were resuspended in YPD broth at an OD_600_ of <0.01. Three separate cultures of each strain were incubated with shaking at either 30 or 20° to OD_600_ ∼0.6. The doubling time (*t*_2_) was calculated for each culture based on the following equation (Amberg *et al.* 2005), where *t_f_* is the time in hours that the cultures were incubated, OD*_f_* is the OD_600_ of the cultures after incubation, and OD*_i_* is the OD_600_ of the dilutions before incubation:

$$t_{2}=t_{f}\left[\frac{\log_{10}2}{\ln\left(\frac{{\text{OD}}_{f}}{{\text{OD}}_{i}}\right)}\right].$$

For serial dilution growth assays, cells from fresh patches on YPD plates were resuspended in YPD broth at an OD_600_ of ∼0.3. These suspensions were diluted 10-fold serially, from 1:10 to 1:10,000. A 5 µl aliquot of each dilution was plated onto YPD agar, with or without 5 µg/ml tunicamycin (Sigma), and incubated at 30° for the indicated times.

### Polysome analysis

Cells were grown to an OD_600_ of 0.4–0.6 at 20° in YPD broth, and 2.0 OD_600_ units of cells were collected. Cells were washed and lysed by bead beating in 20 mM Tris-HCl pH 8.0, 140 mM KCl, 5 mM MgCl_2_, 0.5 mM DTT, 1% Triton X-100, 1 mg/ml heparin, cOmplete Mini, EDTA-free protease inhibitor cocktail (Roche), and 200 units/ml RNasin (Promega). Debris was pelleted at 4000 rpm at 4° for 5 min. Clarified lysates were transferred to a fresh tube, and centrifuged at 10,000 rpm at 4° for 5 min. Cell extracts were separated by ultracentrifugation at 35,000 rpm in 10–50% (w/v) sucrose gradients containing 20 mM Tris-HCl pH 8.0, 140 mM KCl, 5 mM MgCl_2_, 0.5 mM DTT, and 1 mg/ml heparin for 165 min at 4° in an SW41 rotor. Gradients were fractionated with a Teledyne/ISCO gradient fractionation system. Traces were recorded on a UA6 detector at a sensitivity setting of 0.2. In addition, traces for *rpl7a∆ RPL7B* and *rpl7a∆ rpl7b∆*::*RPL7A* strains were collected at a sensitivity setting of 0.1.

### Fluorescence microscopy

Three independent transformants of strains JC3212, JC5987, JC5989, and JC5914 with plasmids p*GAL1_P_-ASH1*-MS2L(x12) and pMS2-CP-GFP(x3) were grown overnight in SC-HIS-LEU broth at 30°. Aliquots of each culture were inoculated into 10 ml of fresh SC-HIS-LEU broth containing 2% galactose, 2% raffinose, and 2% sucrose, and incubated at 30° to OD_600_ of 0.6. A 2.5 OD_600_ unit aliquot of each culture was harvested, resuspended in 1 ml of water, and incubated with 2.5 µl Hoechst 33258 for 20 min at room temperature. Cells were washed three times with water prior to visualization.

Detection of Ty1 RNA by fluorescence *in situ* hybridization was performed as previously described (Doh *et al.* 2014). A Zeiss Axioskop 200 M inverted microscope was used (filter set: 31 for Cy3, and 34 for DAPI) at a magnification of 63×. Photographs were taken with a Q Imagining Camera (or Hamamatsu ORCA ER), and merged and colored with Openlab 4.0.4 software (Improvision).

### Gag-GFP activity assay

Two independent cultures of strain JC3807 and derivatives, which harbor the Ty1(*GAG-GFP*)-3566 chromosomal element (Scholes *et al.* 2003), and strain BY4741 and derivatives, which do not, were grown in YPD broth overnight at 30°. Duplicate cultures of each strain were diluted to an OD_600_ of 0.1 in YPD broth, and grown at 20° for 3 hr. The cells were diluted 1:10 in deionized H_2_O. The geometric mean of the GFP activity in 10,000–20,000 cells of each strain was measured by flow cytometry using a FACSCalibur (Becton Dickinson). The geometric mean of the GFP activity for each strain lacking Ty1(*GAG-GFP*)-3566 was subtracted from the mean GFP activity in the isogenic strain harboring Ty1(*GAG-GFP*)-3566 to correct for autofluorescence arising from differences in cell size.

### RNA isolation and quantitative real-time PCR

Cells of each strain grown overnight in YPD broth at 30° were diluted to an OD_600_ of 0.3 in 50 ml YPD broth, and grown to an OD_600_ of 0.6 at 20°. Cell harvesting, RNA extraction, and DNase treatment was performed as previously described (Risler *et al.* 2012). Total RNA was used as a template for qRT-PCR, which was performed as described previously (Risler *et al.* 2012), with the following modifications. Ty1 and *snR6* RNAs were reverse transcribed with the sequence-specific primers TY5253A and PJ751, respectively. Ty1 and *snR6* single-stranded cDNAs were amplified via qPCR with PJ748 and PJ1230, and PJ750 and PJ1220, respectively. The average fold change in Ty1 RNA level was calculated according to the 2^−∆∆Ct^ method using average Ty1 and *snR6* cycle thresholds (Ct) from three biological replicates.

### Ty1his3AI retrotransposition assays

To determine the retromobility rate of the Ty1*his3AI-∆1-3114* element (Mou *et al.* 2006), three biological replicates of each strain were inoculated to a density of 1 × 10^−4^ cells/ml in seven cultures of YPD broth for a total of 21 cultures per genotype. Cultures were incubated with shaking at 20° for 3–4 d. A 1 µl aliquot of a 1 × 10^−3^ dilution of four randomly selected cultures was plated on SC agar to determine the titer. All seven cultures were plated on SC-HIS agar to determine the number of His^+^ prototrophs. The Ty1*his3AI-∆1* retromobility rate for each genotype was determined by dividing the mean titer into the mutation rate, which was calculated by using the median number of His^+^ prototrophs per 1 ml culture in the Lea and Coulson median estimator (Rosche and Foster 2000).

To determine the frequency of Ty1*his3AI-∆1-3114* retrotransposition during induction of expression from plasmid pGTy1(*synGAG*)∆3′LTR, four independent transformants of strains harboring plasmid pRS415, or pGTy1(*synGAG*)∆3′LTR, were inoculated into SC-LEU broth containing 2% raffinose and 2% sucrose, and incubated overnight at 30° with rolling. Cultures were diluted 1:10 in YP broth containing 2% galactose, 2% raffinose, and 2% sucrose, and incubated at 20° for 3 d. A 1 µl aliquot of each culture was plated on SC-LEU agar to determine the number of cells that retained the plasmid throughout incubation, and the remainder of each culture was plated on SC-LEU-HIS agar to determine the number of His^+^ prototrophs. The mean Ty1*his3AI* retromobility frequency is the mean of the number of His^+^Leu^+^ prototrophs per culture divided by the total number of Leu^+^ prototrophs per culture.

To determine the retromobility frequency of the Ty1*his3AI-∆1-3114* element in congenic *RAD52* and *rad52* strains, seven independent cultures of yeast cells of each genotype were inoculated to a density of 1 × 10^−4^ cells/ml in YPD broth, and cultures were grown at 20° each genotype were inoculated to a density^−6^ dilution of each culture was plated on SC agar to determine the titer. The remaining culture was plated on SC-HIS agar to determine the number of His^+^ prototrophs. The retromobility frequency of each culture was calculated as the fraction of total cells that were His^+^. The mean retromobility frequency was determined from seven cultures of each genotype.

### Quantitative Southern blot assay for Ty1 cDNA

To quantify unintegrated Ty1 cDNA in each strain, total genomic DNA was harvested from cells grown to saturation at 20°. DNA samples were digested with *Sph*I, and separated on a 1% TBE agarose gel. Southern blot analysis was performed with an antisense Ty1 *POL* riboprobe *in vitro*-transcribed using plasmid pJC525 as a template. The intensity of bands corresponding to Ty1 cDNA, and two different genomic DNA bands, was measured with ImageQuant software.

### Data availability

Strains and plasmids are available upon request. Figure S1 illustrates nucleotide substitutions in Ty1(*syn-Gag*) relative to Ty1-H3. Table S1 contains genotypes of strains used in this study. Table S2 contains oligonucleotide primers used in this study. Table S3 lists the chromosomal location and orientation of Ty1*HIS3* transposition events. File S1 contains methods and references for Table S3. All data necessary for confirming the conclusions presented in the article are represented fully within the article and the associated supplementary files.

## Results

### Construction of chimeric RPL7 alleles

To determine whether the Rpl7 or snoRNA isoforms encoded by *RPL7A* and *RPL7B* have evolved distinct functions irrespective of the disparity in their expression, we created chimeric *RPL7* alleles in which the CRI of one paralog, extending from the start codon to the stop codon, and including the *snR39* or *snR59* snoRNA gene, was substituted for the CRI of the other paralog. The resulting chimeric alleles consist of the CRI of one paralog, and the 5′ UTR, 3′ UTR, promoter, and terminator, denoted as the “locus,” of the other. Mutants harboring a chimeric allele were constructed by precisely replacing the CRI of *RPL7A* or *RPL7B* in strain JC3212 with a counterselectable *delitto perfetto* cassette (Storici and Resnick 2006) (Figure 1B). Each cassette was then precisely replaced with the *RPL7B* or *RPL7A* CRI, respectively. The resulting “homogenic” strains harbor the same CRI at both the native allele, and a chimeric *rpl7b∆*::*RPL7A* or *rpl7a∆*::*RPL7B* allele (Clarkson *et al.* 2010). Subsequently, the *RPL7A* or *RPL7B* CRI was deleted at its native locus in homogenic strains to yield “chimeragenic” strains (Figure 1B). Since one *RPL7* gene is required for survival (Mizuta *et al.* 1992), the viability of chimeragenic strains demonstrates that both chimeric *RPL7* alleles are functional.

### Growth rates and 60S ribosomal subunit abundance in strains with different RPL7 alleles

**Figure 2:**
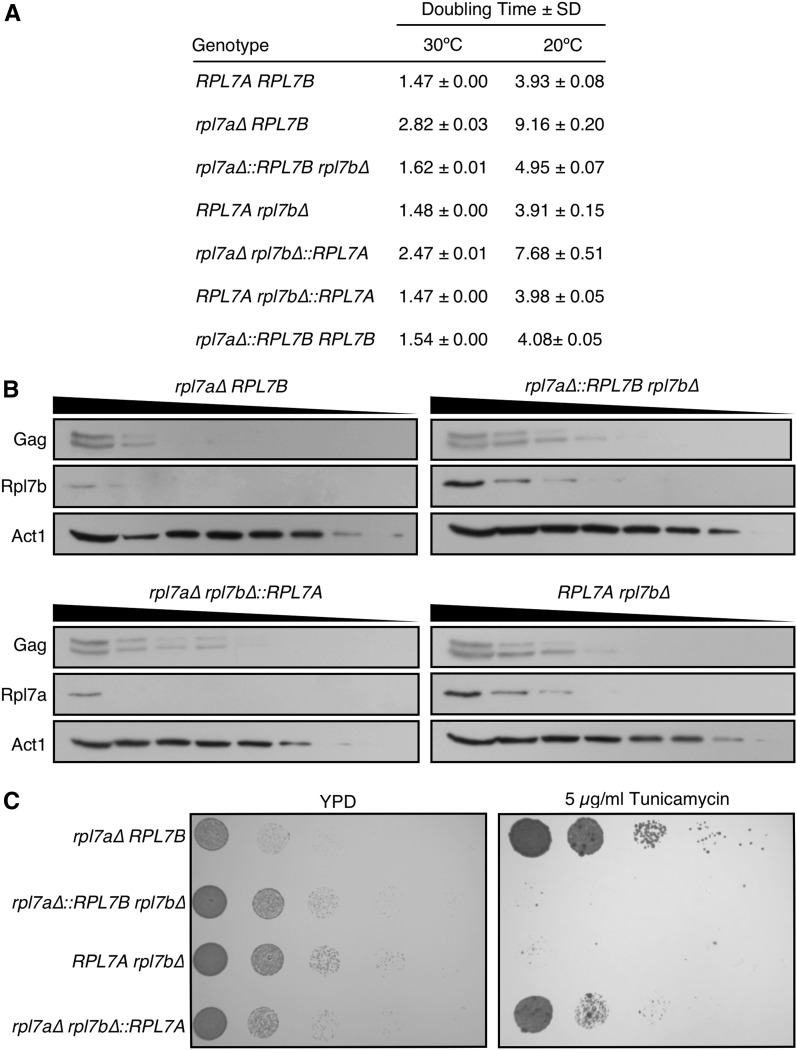
The *RPL7* locus is the primary determinant of cell growth rate, Rpl7 level and tunicamycin resistance. (A) The rate of cell doubling ± SD in three biological replicates of each strain of the indicated genotype grown in YPD broth at 20 or 30° to an OD_600_ of 0.6. (B) Western blot analysis of Rpl7 and Ty1 Gag, which runs as a doublet, in *rpl7* deletion and chimeragenic strains. Each lane from left to right represents a twofold serial dilution of whole cell lysate. Gag was detected with anti-VLP antisera, and Rpl7 isoforms were detected with polyclonal anti-human L7 antibodies. Act1 was detected with a monoclonal anti-Actin antibody as a loading control. (C) The relative tunicamycin resistance of each strain of the indicated genotype grown to an OD_600_ of 0.6 in YPD broth at 30°. A 5 µl aliquot of each culture, and 1:10 serial dilutions were plated on YPD agar, with or without 5 µg/ml tunicamycin. Plates were incubated at 30° for 1 d (YPD) or 5 d (YPD with tunicamycin).

The growth rates in *RPL7* deletion, chimeragenic and homogenic strains were measured in YPD broth at 30 and 20°, the latter permissive for retrotransposition of Ty1 (Figure 2A). The *RPL7Arbl7b∆* mutant had a wild-type growth rate. Mutants expressing the *RPL7A* or *RPL7B* CRI from the *RPL7B* locus exhibited doubling times that were 1.7-fold longer at 30°, and 1.9- to 2.0-fold longer at 20°, when compared to strains expressing the corresponding CRI from the *RPL7A* locus. These data suggest that, in mutants harboring only one *RPL7* allele, the *RPL7A* locus is more strongly expressed than the *RPL7B* locus. There was also a small increase in doubling time (1.1-fold longer at 30° and 1.2- to 1.3-fold longer at 20°) when the *RPL7B* *vs.* the *RPL7A* CRI was expressed from either locus, suggesting that higher levels of Rpl7a/snR39 are expressed from either *RPL7* loci, or that Rpl7a/snR39 is more active than Rpl7b/snR59 in 60S ribosomal subunit biogenesis. Increasing the dosage of Rpl7b/snR59 by expressing the *RPL7B* CRI from both loci suppressed the major and minor growth defect of mutants expressing the *RPL7B* CRI from the *RPL7B* or *RPL7A* locus, respectively. In fact, the doubling times of the homogenic *rpl7a∆*::*RPL7B* strain at 20 and 30° were within 5% of those of the wild-type strain, and those of the homogenic *RPL7Arpl7b∆*::*RPL7A* strain were indistinguishable from wild type. These findings indicate that the *RPL7* locus or loci expressed have a greater effect on cell doubling time than the particular *RPL7* CRI that is expressed.

**Figure 3:**
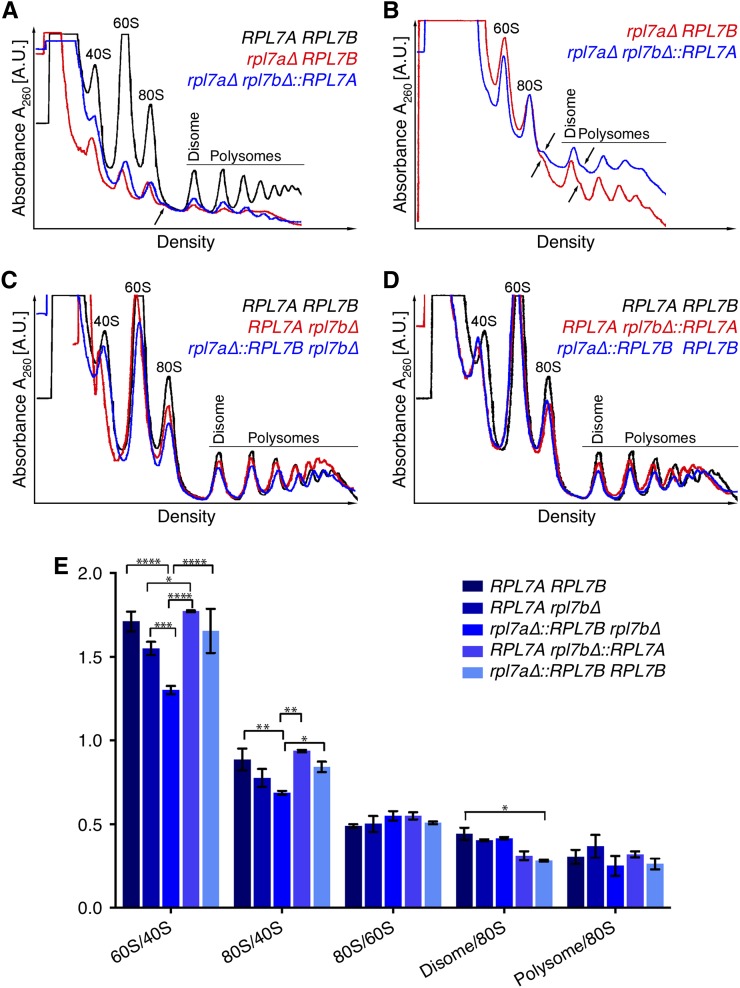
Primarily the *RPL7* locus, but also the Rpl7 and snR39/59 isoforms expressed, contributes to differences in polysome profiles. Polysome profiles of cell extracts from strains of the indicated genotype grown in YPD broth at 20°, and fractionated in 10–50% (w/v) sucrose gradients. Absorbance units at 254 nm from the top to the bottom of the gradient for each genotype are shown in each graph. The same trace of the *RPL7A RPL7B* strain is displayed in black in (A), (C), and (D) to facilitate comparisons between panels. (A) Polysome profiles of the mutants with the *RPL7B* (red), or *RPL7A* (blue), CRI at the *RPL7B* locus are shown, and the genotypes indicated. Traces of the two mutants and the *RPL7A RPL7B* strain were overlaid by aligning the lowest point of each trace between the 80S monosome peak and the disome peak. The sensitivity setting of the UV monitor was 0.2. The arrow points to a minor peak of halfmer polyribosomes on the right shoulder of the 80S peak in *rpl7a∆ RPL7B* and *rpl7a∆ rpl7b∆*::*RPL7A* mutants. (B) Reanalysis of the *rpl7a∆ RPL7B* and *rpl7a∆ rpl7b∆*::*RPL7A* mutants, with the UV monitor at a more sensitive setting of 0.1, and profiles normalized to the 80S peak for better resolution of the halfmer polyribosome peaks, which are indicated by arrows. The 40S peak cannot be resolved at this sensitivity setting. (C, D) Polysome profiles of mutants of the indicated genotypes with the *RPL7A* (red), or *RPL7B* (blue), CRI at the *RPL7A* locus. In each panel, traces of three strains were overlaid by aligning the lowest point of each trace between the 80S monosome peak and disome peak. The sensitivity setting of the UV monitor was 0.2. (E) A graph of relative levels of 60S ribosomal subunits, 80S monosomes, disomes, and polysomes in three biological replicates of each strain of the indicated genotype. To normalize for differences in lysis efficiency and lysate amount, the heights of 40S, 60S, 80S, disome, and highest polysome peaks in polysome profiles were measured, and ratios indicated on the *y*-axis were calculated. Statistically significant differences in the indicated ratios between strains of different genotypes, as determined by a two-way ANOVA test, are indicated with asterisks. *P < 0.05, **P < 0.01, ***P < 0.001, ****P < 0.0001.

Polysome profiles of the *RPL7* chimeragenic and deletion strains were compared to determine whether differences in cell doubling times are reflected in altered 60S ribosomal subunit levels (Figure 3). Cells were grown at 20° because differences in doubling time were more pronounced at this temperature (Figure 2A). Even the wild-type strain had higher 40S and 60S ribosomal subunit peaks than 80S or polysome peaks at this suboptimal temperature (Figure 3A). Nonetheless, mutants with either *RPL7* CRI at the *RPL7B* locus only had greatly diminished levels of 60S ribosomal subunits, 80S monosomes, and polysomes relative to the wild-type strain (Figure 3A). In addition, halfmer peaks, in which 40S ribosomal subunits that are not joined with 60S subunits are associated with mRNA, were evident as shoulders on the 80S monosome and disome peaks (Figure 3B), indicating that the ratio of 60S to 40S ribosomal subunits is markedly reduced in these mutants. In contrast, the polysome profiles of mutants with either *RPL7* CRI at the *RPL7A* locus more closely resembled that of the wild-type strain, and halfmer polysomes were not detected (Figure 3C). Compared to 40S ribosomal subunits, there was a significantly lower level of 60S ribosomal subunits, and reduced 80S monosomes and polysomes when the *RPL7B* CRI *vs.* the *RPL7A* CRI was present at the *RPL7A* locus (Figure 3, C and E). These discrepancies paralleled the modest difference in growth rate between these mutants. Expression of the *RPL7B* CRI from both *RPL7* loci suppressed the 60S/40S ribosomal subunit imbalance of the *rpl7a∆*::*RPL7B∆* mutant (Figure 3, C**–**E). The polysome profiles of homogenic*RPL7Arpl7b∆*::*RPL7A* and *rpl7a∆*::*RPL7B* strains were both similar to that of the wild-type strain, although there was a minor reduction in light polysomes in both (Figure 3D), and a decrease in the ratio of disomes to 80S monosomes in the *rpl7a∆*::*RPL7B* (Figure 3E).

It was not possible to determine whether the 60S to 40S ribosomal subunit ratio differed in mutants with the *RPL7B* *vs.* *RPL7A* CRI at the *RPL7B* locus, since the level of 40S subunits was so low in both strains (Figure 3A). Nonetheless, the polysome data from mutants expressing *RPL7* CRI from the *RPL7A* locus suggests that a reduced ratio of 60S to 40S ribosomal subunits is strongly correlated with differences in the growth rate of *rpl7* deletion and chimeragenic strains.

To determine whether doubling time and 60S ribosomal subunit level differences among strains expressing only one native or chimeric *RPL7* allele were reflected in differences in Rpl7 levels, western blot analysis of twofold serial dilutions of cell lysate with a human anti-L7 antibody that recognizes the identical epitope in Rpl7a and Rpl7b was performed. Rpl7 levels relative to level of a control protein, Act1 were measured by densitometry using bands at nonsaturating intensities. The Rpl7/Act1 ratio was >2-fold higher when the *RPL7A* CRI was expressed from the *RPL7A* locus *vs.* the *RPL7B* locus (0.045 *vs.* 0.020, respectively), or when the *RPL7B* CRI was expressed from the *RPL7A* locus *vs.* the *RPL7B* locus (0.029 *vs.* 0.012, respectively) (Figure 2B). In addition, there was a modestly higher level of Rpl7 when the *RPL7A* CRI *vs.* the *RPL7B* CRI was expressed from either the *RPL7A* locus (0.045 *vs.* 0.029) or the *RPL7B* locus (0.020 *vs.* 0.012). Therefore, differences in growth rates and relative 60S ribosomal subunit levels among strains expressing a single native or chimeric *RPL7* allele may result, at least partially, from different Rpl7 levels. Together these findings indicate that *RPL7Arpl7b∆* > *rpl7a∆*::*RPL7B∆* > *rpl7a∆ rpl7b∆*::*RPL7A > rpl7a∆ RPL7B* for rate of growth, ratio of 60S to 40S ribosomal subunits, and Rpl7 level.

### Negative correlation between tunicamycin resistance and Rpl7 and snR39/59 expression

Deletion of *RPL7A*, but not *RPL7B*, results in resistance to tunicamycin, an inhibitor of protein N-glycosylation in the endoplasmic reticulum (ER), and inducer of the ER stress response (Steffen *et al.* 2012). We asked whether resistance to tunicamycin is a function of expressing the *RPL7B* CRI or simply correlated with Rpl7 levels by plating serial dilutions of *rpl7* deletion and chimeragenic strains on YPD agar, with or without 5 µg/ml tunicamycin (Figure 2C). Mutants with either *RPL7* CRI at the *RPL7A* locus were sensitive to tunicamycin, while mutants with either *RPL7* CRI at the *RPL7B* locus were resistant (Figure 2C). There was a small decrease in tunicamycin-resistance in the mutant with the *RPL7A* *vs.* the *RPL7B* CRI at the *RPL7B* locus (Figure 2C), which parallels the slightly faster growth and higher Rpl7 level in the *rpl7a∆ rpl7b∆:RPL7A* mutant (Figure 2, A and B). Together, the data suggest that tunicamycin resistance is correlated with Rpl7 and snR39/59 levels, and not a specific function of *RPL7B* isoforms.

### ASH1 mRNA localization in mutants with an RPL7A or rpl7a∆::RPL7B allele

**Figure 4:**
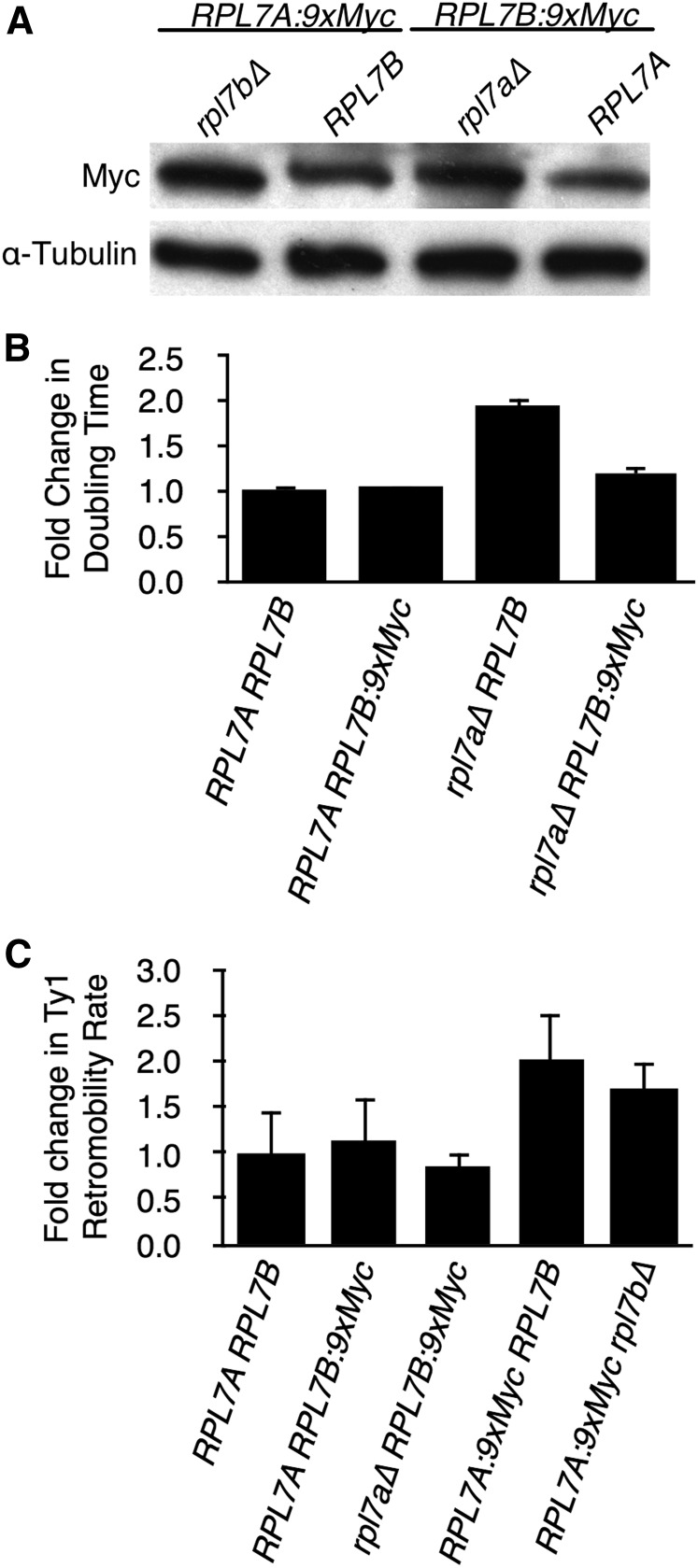
An epitope-tagged *RPL7B* allele suppresses the growth and Ty1 retromobility defect of an *rpl7a∆* mutant. (A) Western blot analysis of Rpl7a-9xMyc or Rpl7b-9xMyc in whole cell lysate from strains, without or with deletions of *RPL7B* or *RPL7A*, respectively. Rpl7a-9xMyc and Rpl7b-9xMyc were detected with anti-c-Myc antibody, and α-Tubulin was detected with an anti-α-Tubulin antibody as a loading control. (B) The rate of cell doubling of three biological replicates of each strain of the indicated genotype grown in YPD broth at 20° to an OD_600_ of 0.4–0.6. Error bars represent SD. (C) The fold-change in retrotransposition rate is the retrotransposition rate of a chromosomal Ty1*his3AI* element in each strain of the indicated genotype relative to that in the *RPL7A RPL7B* strain. Error bars represent SE.

The *ASH1* mRNA transcript localizes to a focus at the tip of the budding daughter cell during bud growth, and migrates toward the bud neck as the cell progresses through M-phase (Beach *et al.* 1999). Deletion of *RPL7A* but not *RPL7B* significantly reduces the percentage of budded cells with bud-localized *ASH1* mRNA foci (Komili *et al.* 2007). To determine whether the *RPL7A* isoforms or locus contribute to *ASH1* mRNA localization to the bud, an mRNA consisting of the *ASH1* ORF and 12 MS2 stem-loops [*ASH1-MS2L(x12)*] was expressed in *rpl7* chimeragenic and deletion strains. Coexpression of an MS2 coat protein, which binds to the MS2L(x12) RNA sequences, fused to three GFP moieties [MS2-CP-GFP(x3)] was used to detect *ASH1-MS2L(x12)* RNA by fluorescent microscopy of live cells. As expected, a lower percentage of budded *rpl7a∆ RPL7B* cells had a bud-localized *ASH1-MS2L(x12)* mRNA focus compared to budded *RPL7Arpl7b∆* or wild-type cells (Figure 4). An equivalently low level of bud-localized *ASH1-MS2L(x12)* mRNA was seen when *RPL7A* was expressed from the *RPL7B* locus (*rpl7a∆ rpl7b∆*::*RPL7A*, Figure 4), indicating that *ASH1* mRNA localization is inefficient when either *RPL7* CRI is expressed from the *RPL7B* locus. In contrast, expression of either CRI from the *RPL7A* locus resulted in the same percentage of budded cells exhibiting bud-localized *ASH1-MS2L(x12)* mRNA as in the wild-type strain. (Compare *RPL7Arpl7b∆*, *rpl7a∆*::*RPL7B∆*, and *RPL7ARPL7B*, Figure 4.) Together, these data suggest that either the *RPL7A* or *RPL7B* CRI, when expressed from the *RPL7A* locus results in sufficient levels of Rpl7 and snR39/59 to support wild-type levels of *ASH1* mRNA localization.

### Ty1 retromobility is a function of robust Rpl7 and Rpl31 expression

Ty1 retromobility is strongly reduced in the absence of *RPL7A*, or a specific paralog of a few other RP gene pairs (Risler *et al.* 2012). Retrotransposons have the potential to damage the host genome when their rate of mobility or location of insertion into the genome is not tightly controlled; therefore, we considered the possibility that differences between Rpl7b/snR59 and Rpl7a/snR39 isoforms might confer a selective advantage on the cell by repressing Ty1 retromobility while maintaining ribosome-related functions. To determine whether Ty1 retromobility is a paralog-specific function of *RPL7A*, we used a chromosomal Ty1*his3AI* element to measure retromobility in *RPL7* deletion strains. The *his3AI* retrotranscript indicator gene contains an antisense intron interrupting the *HIS3* coding sequence. The *his3AI* reporter is located in the 3′ UTR of the chromosomal Ty1 element in the opposite transcriptional orientation; ergo, it can be spliced from the Ty1 transcript. Cells in which Ty1*his3AI* RNA is spliced and reverse transcribed, and the resulting Ty1*HIS3* cDNA is inserted into the genome by integration or recombination, are detected as His^+^ prototrophs. The rate of His^+^ prototroph formation per cell per generation is directly proportional to the rate of Ty1*his3AI* retromobility (Curcio and Garfinkel 1991). At 20°, a permissive temperature for Ty1 retrotransposition, Ty1*his3AI* retromobility in the *rpl7a∆ RPL7B* mutant was <8% of that in the wild-type strain, but in an *RPL7Arpl7b∆* mutant, the rate of retromobility was equivalent to that in a wild-type strain (Table 1). Thus, Ty1 retromobility is a paralog-specific function of *RPL7A*.

**Table 1:** Effect of *RPL7* and *RPL31* alleles on Ty1*his3AI* retromobility

Genotype	Rate of Ty1*his3AI* Retromobility*^a^* ± SE × 10^−7^	Relative Ty1*his3AI* Retromobility Rate
*RPL7A RPL7B*	1.01 ± 0.23	1.0
*rpl7a∆ RPL7B*	<0.08	<0.08
*RPL7A rpl7b∆*	1.07 ± 0.20	1.1
*rpl7a∆ rpl7b∆*::*RPL7A*	0.13 ± 0.03	0.1
*rpl7a∆*::*RPL7B rpl7b∆*	0.82 ± 0.18	0.8
*rpl7a∆*::*RPL7B RPL7B*	0.77 ± 0.26	0.8
*RPL7A rpl7b∆*::*RPL7A*	0.82 ± 0.21	0.8
*RPL31A RPL31B*	0.68 ± 0.29	1.0
*rpl31a∆ RPL31B*	0.03 ± 0.05	0.05
*rpl31a∆*::*RPL31B rpl31b∆*	0.79 ± 0.35	1.2

a Median number of His^+^ prototrophs per cell per generation.

The rate of Ty1*his3AI* retromobility in the *rpl7a∆*::*RPL7B∆* mutant was similar to that of the *RPL7Arpl7b∆* strain, suggesting that Rpl7a/snR39 isoforms do not play a specialized role in Ty1 retrotransposition. This conclusion is also supported by the observation that homogenic mutants expressing either the *RPL7A* or the *RPL7B* CRI at both loci had high levels Ty1 retromobility that were comparable to each other, and similar to the wild-type strain. In contrast, the *rpl7a∆ rpl7b∆*::*RPL7A* mutant had a strongly reduced retromobility rate similar to that of the *rpl7a∆ RPL7B* mutant (Table 1). Thus, expression of either Rpl7a/snR39 or Rpl7b/snR59 isoforms from the *RPL7A* locus, but not the *RPL7B* locus, resulted in efficient Ty1 retromobility.

**Figure 5:**
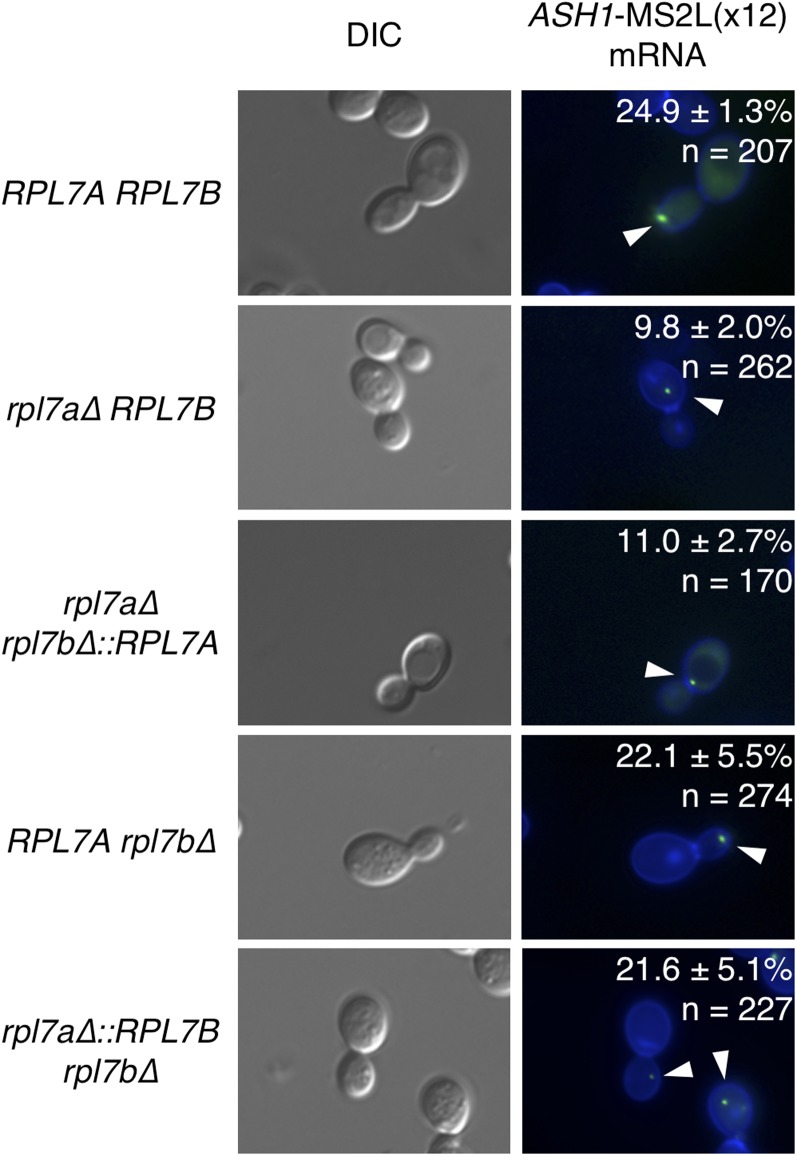
*ASH1* mRNA localization requires a high dosage of Rpl7 and snR39/59. Expression of *ASH1*-MS2L(x12) RNA was induced in strains of the indicated genotype cotransformed with plasmid pGAL1_P_*ASH1*-MS2L(x12), and plasmid pMS2-CP-GFP(x3), by growth in galactose-containing medium. The *ASH1* ORF fragment in *ASH1*-MS2L(x12) RNA contains three sequence motifs—E1, E2 and E3—that target the mRNA for localization to the bud tip (Beach and Bloom 2001). Cells were incubated with Hoechst 33258 dye, which stains the yeast cell wall (Hernandez *et al.* 1988). Cells from two or three biological replicates were analyzed for a total of three independent experiments. Numerical values represent the average percent of budded cells with bud-localized *ASH1*-MS2L(x12) mRNA foci; *n* represents the total number of budded cells counted. White arrows point to *ASH1*-MS2L(x12) RNA foci in budded cells.

To further test the idea that the frequency of Ty1 retromobility is correlated with the level of Rpl7, we capitalized on an interesting observation: when *RPL7B* is tagged with a *9xMYC* epitope at the 3′ end of the CRI, the level of Rpl7b:9xMyc increased upon deletion of *RPL7A* (Figure 5A). Addition of the *9xMYC* tag to *RPL7B* did not change the doubling time of cells with an *RPL7A* allele, but it substantially decreased the doubling time of an *rpl7a∆* mutant (Figure 5B). Thus, in contrast to the ∼2-fold difference in doubling time in an *rpl7a∆ RPL7B* mutant relative to the *RPL7ARPL7B* strain (Figure 2A and Figure 5B), there was a very slight difference in doubling time between the *rpl7a∆ RPL7B:9xMYC* mutant and *RPL7ARPL7B:9xMYC* strain (Figure 5B), consistent with the compensatory increase in Rpl7b:9xMyc protein when *RPL7A* was deleted. Also, there was no significant difference in the frequency of Ty1*his3AI* retromobility between the *rpl7a∆ RPL7B:9xMYC* mutant and *RPL7ARPL7B:9xMYC* strain (Figure 5C), which contrasts starkly with the >10-fold difference in Ty1*his3AI* retromobility between *rpl7a∆ RPL7B* and *RPL7ARPL7B* strains (Table 1). The data suggest that increased expression of Rpl7b:9xMyc upon deletion of *RPL7A* suppresses the retromobility defect that normally results from deleting *RPL7A*, and strongly support the idea that the *RPL7A* paralog is required for retromobility because of the level of Rpl7 expression rather than the expression of the Rpl7a or snR39 isoform.

The *RPL31A* gene, but not *RPL31B*, was also identified as essential for efficient Ty1 retromobility (Risler *et al.* 2012). *RPL31A* and *RPL31B* ORFs are not interrupted by introns, and encode isoforms that differ at only one amino acid residue. The rate of Ty1*his3AI* retromobility in an *rpl31a∆ RPL31B* mutant was 5% of that in a wild-type strain (Table 1). Ty1*his3AI* retromobility in a chimeragenic strain expressing the *RPL31B* ORF from the *RPL31A* locus was measured to determine whether Rpl31 dosage or the Rpl31a isoform is required for Ty1 retromobility. The *rpl31a∆*::*RPL31B∆* strain had a similar rate of Ty1 retromobility to that of the wild-type strain, and a 23-fold higher rate than the *rpl31a∆ RPL31B* mutant (Table 1), indicating that expression of either Rpl31 isoform from the *RPL31A* locus is sufficient for Ty1 retromobility. Since deletion of *RPL31A* but not *RPL31B* substantially retards cell doubling (Steffen *et al.* 2012), a higher dosage of Rpl31b when expressed from the *RPL31A* *vs.* *RPL31B* locus is the most plausible cause of more efficient Ty1 retromobility.

### Minor effects of Rpl7 and snR39/59 levels on Ty1 RNA translation

**Figure 6:**
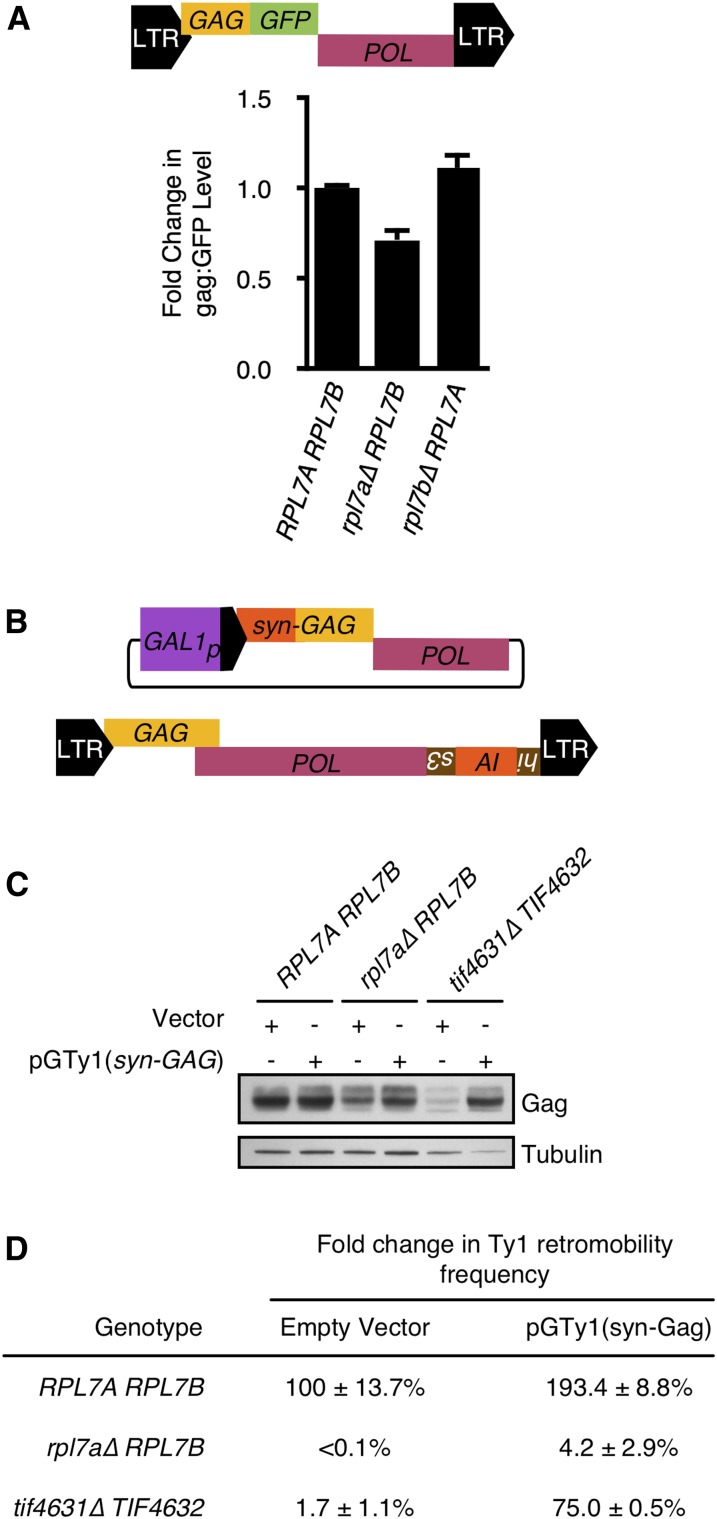
Diminished Ty1 retromobility in the *rpl7a∆ RPL7B* mutant is not a result of limiting Gag levels. (A) A schematic of the chromosomal Ty1(*GAG:GFP*)-3566 element is shown (top). The *GFP* ORF is fused at the C-terminal end of the *GAG* ORF after codon 401 in a chromosomal Ty1 element (Scholes *et al.* 2003). The mean fluorescence in 10,000–20,000 cells of each genotype, corrected for autofluorescence by subtracting the mean fluorescence in each congenic strain lacking Ty1(*GAG:GFP*)-3566 from the mean fluorescence in each strain harboring Ty1(*GAG:GFP*)-3566, was determined, and the value relative to that in the *RPL7A RPL7B* strain is reported (bottom panel). Error bars represent the SD of mean fluorescence calculated from two independent isolates of each genotype. (B) Schematic of plasmid pGTy1(*syn-GAG*), which carries a modified Ty1 element that lacks the 3′ LTR, and has numerous silent nucleotide substitutions in the *GAG* ORF. Ty1(*syn-GAG*) RNA is defective for retromobility in *cis*, but encodes wild-type Gag and Gag-Pol proteins required in *trans* for retromobility of the chromosomal Ty1*his3AI* element, which is diagrammed below plasmid pGTy1(*syn-GAG*). (C) Western blot analysis of *RPL7A RPL7B*, *rpl7a∆ RPL7B*, and *tif4631∆ TIF4632* strains harboring either the vector pRS415 or plasmid pGTy1(*syn-GAG*) and grown in galactose medium at 20°. The membrane was probed with anti-VLP antisera to detect Ty1 Gag (top), or with an anti-α-Tubulin antibody to detect α-Tubulin (bottom) as a loading control. (D) Effect of pGTy1(*syn-GAG*) expression on Ty1*his3AI* retromobility in different genetic backgrounds. *RPL7A RPL7B*, *rpl7a∆ RPL7B* and *tif4631∆ TIF4632* strains harboring a chromosomal Ty1*his3AI* element, and the vector (pRS415), or plasmid pGTy1(*syn-GAG*) grown in galactose-containing medium at 20° to induce expression of Ty1 proteins, and the Ty1 retromobility frequency was determined by measuring the frequency of His^+^ prototrophs formed. Error represents the SD of four biological replicates.

Depletion of 60S ribosomal subunits in mutants with low levels of Rpl7 could inhibit Ty1 retromobility by selectively reducing the rate of Ty1 RNA translation. However, Gag levels were not significantly different among chimeragenic and deletion mutants harboring one *RPL7* allele (Figure 2B). The *rpl7a∆ RPL7B* mutant had 59% as much Gag as the *RPL7Arpl7b∆* mutant, despite substantial differences in doubling time and 60S subunit levels between these strains (Figure 2, A and B). As an independent method of measuring relative Gag levels in the *rpl7a∆ RPL7B* mutant, we quantified Gag:GFP produced from a chromosomal Ty1(*GAG:GFP*) element (Figure 6A, top). The *rpl7a∆ RPL7B* mutant had ∼75% of the GFP activity in the wild-type strain or *RPL7Arpl7b∆* mutant (Figure 6A, bottom). Thus, even the mutant with the strongest depletion of Rpl7 and 60S ribosomal subunits has only a minor defect in Gag accumulation, indicating that inhibition of Ty1 RNA translation may not be the major cause of the retromobility defect in the *rpl7a∆ RPL7B* mutant.

To further determine whether the retromobility defect of the *rpl7a∆ RPL7B* mutant is a direct result of limiting levels of Ty1 proteins, the ability of Ty1 proteins supplied in *trans* to suppress the retromobility defect was tested. Ty1 proteins were expressed from a *GAL1*-driven modified Ty1 element whose transcript cannot be used as a template for retrotransposition. The plasmid-borne pGTy1(*syn-GAG*) element lacks a 3′ LTR, and has multiple nucleotide substitutions in the 5′ UTR and 5′ terminus of the *GAG* ORF that are predicted to disrupt RNA secondary structures and other *cis*-acting sequences required for retrotransposition (Curcio *et al.* 2015), but not the amino acid sequence of Gag (Figure 6B and Figure S1). As a positive control for suppression of a Ty1 RNA translation defect, pGTy1(*syn-GAG*) was also expressed in a *tif4631*∆ strain. In the absence of *TIF4631*, which encodes the translation initiation factor eIF4G1, the amount of Ty1 Gag is substantially reduced (Figure 6C, *tif4631∆ TIF4632* strain with vector), presumably because eIF4G1 is required to initiate translation from the highly structured 5′ UTR of Ty1 RNA (Purzycka *et al.* 2013). In the *tif4631*∆ strain, galactose-mediated induction of pGTy1(*syn-GAG*) increased the amount of Gag (Figure 6C, compare *tif4631∆ TIF4632* strain with pGTy1(*syn-Gag*) plasmid to that with vector). Accordingly, retromobility of the chromosomal Ty1*his3AI* element increased to 75% of that of the wild-type strain with a vector only (Figure 6D). In contrast, expression of pGTy1(*syn-GAG*) in the *rpl7a∆ RPL7B* strain increased Gag levels [Figure 6C, compare *rpl7a∆ RPL7B* strain with pGTy1(*syn-GAG*) to that with vector], but it only increased Ty1*his3AI* retromobility to 4.2% of that in the wild-type strain with a vector only (Figure 6D). Together, the data suggest that Gag is not the only limiting factor for retrotransposition in the *rpl7a∆ RPL7B* mutant.

### High levels of Rpl7 and 60S ribosomal subunits are required for Ty1 RNA localization

**Figure 7:**
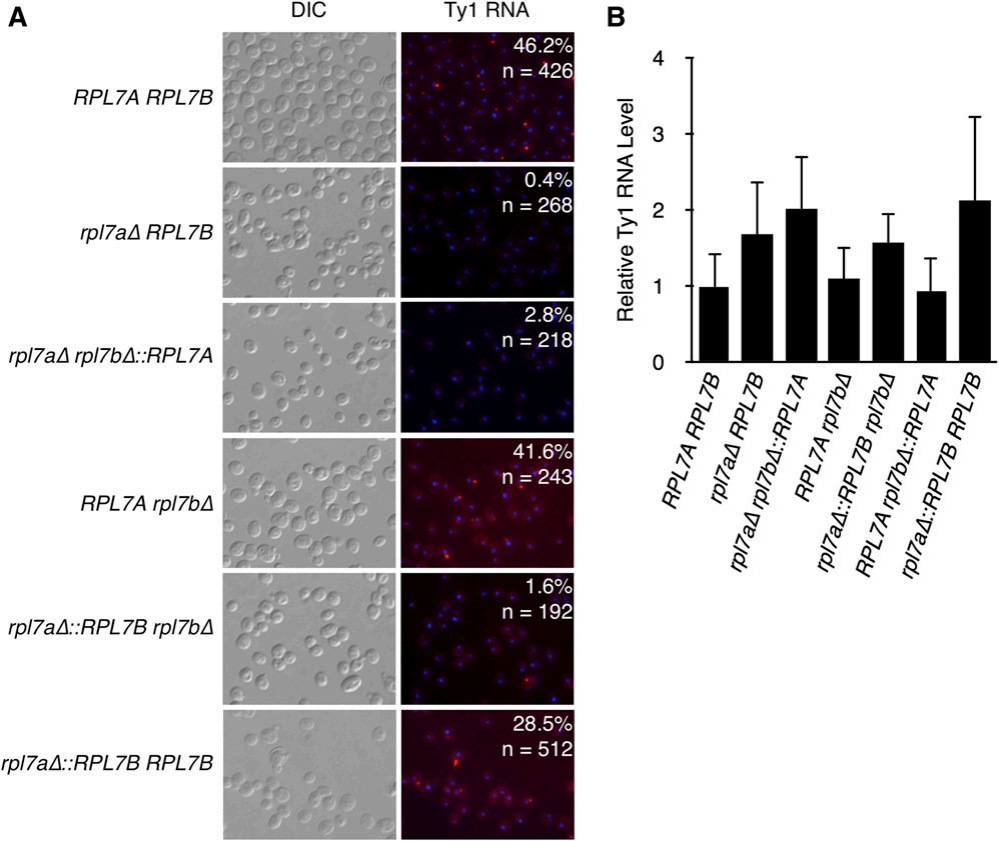
A high dosage of Rpl7 or snR39/59 is required for localization of Ty1 RNA to retrosomes. (A) Fluorescent *in situ* hybridization was used to determine the number of cells harboring a cytoplasmic focus of Ty1 RNA, or retrosome. Ty1 RNA (red) was detected with a Cy3-labeled oligonucleotide probe. Nuclei (blue) were stained with DAPI. The percentage of cells with one or more Ty1 RNA retrosome is indicated. “*n*” is the number of cells scored for the presence of an RNA focus. (B) Quantitative real-time PCR of total RNA to determine the amount of Ty1 RNA relative to the amount of *snR6* RNA in strains of the indicated genotypes. Error bars represent the SD of three biological replicates analyzed in triplicate.

When cells are grown at the permissive temperature for Ty1 retrotransposition, Ty1 RNA is cotranslationally localized to a discrete cytoplasmic focus known as the retrosome, the presumptive site of VLP assembly (Malagon and Jensen 2008; Checkley *et al.* 2010; Sandmeyer and Clemens 2010; Doh *et al.* 2014). Ty1 RNA does not localize in retrosomes in the *rpl7a∆ RPL7B* mutant (Doh *et al.* 2014). To determine whether this localization defect is specific to deleting *RPL7A*, fluorescent *in situ* hybridization (FISH) of Ty1 RNA was performed in strains bearing a deletion of each *RPL7* allele (Figure 7A). Consistent with the previous finding, the percentage of cells with at least one Ty1 RNA focus in an *rpl7a∆ RPL7B* strain was 0.4%; in contrast, 41.6% of *RPL7Arpl7b∆* cells had a Ty1 RNA focus, which was similar to the level in the *RPL7ARPL7B* strain. Therefore, some feature of *RPL7A*, but not *RPL7B*, is required for Ty1 RNA localization to retrosomes. In the mutant with the *RPL7A* CRI expressed from the *RPL7B* locus, the percentage of cells with a Ty1 RNA focus was 2.8%, indicating that expression of either CRI from the *RPL7B* locus is not sufficient for Ty1 RNA localization. Notably, strains expressing the *RPL7B* CRI from the *RPL7A* locus also had a very low percentage of cells with a Ty1 RNA focus (1.6%). Lack of Ty1 RNA localization in these three Rpl7-deficient mutants is not a consequence of reduced Ty1 RNA levels (Figure 7B). Efficient retrosome formation in the *RPL7Arpl7b∆* mutant, but not the *rpl7a∆*::*RPL7B∆* mutant, raises the possibility that a very high level of Rpl7 or snR39/59—one which results in no growth defect or 60S ribosomal subunit deficiency—is necessary for Ty1 RNA to localize to retrosomes. Alternatively, retrosome formation could be a distinct function of the Rpl7a/snR39 isoforms, which must be expressed from the more robust *RPL7A* locus to perform this function efficiently. To differentiate between these possibilities, retrosomes in the homogenic strain expressing the *RPL7B* CRI from both *RPL7* loci were quantified. The percentage of cells with a Ty1 RNA retrosome increased 18-fold relative to the strain expressing the *RPL7B* CRI from the *RPL7A* locus alone, (Figure 7A), indicating that Rpl7b/snR59 supports retrosome formation when expressed at high levels, and, therefore, that Ty1 RNA localization is not a distinct function of the Rpl7a/snR39 isoforms. The finding that Ty1 RNA efficiently localizes to retrosomes only in mutants with growth rates similar to the *RPL7ARPL7B* strain suggests that Ty1 RNA localization is very sensitive to reductions in 60S ribosomal subunits.

### Rpl7 or snR39/59 isoforms influence Ty1 cDNA accumulation in a dosage-dependent manner

**Figure 8:**
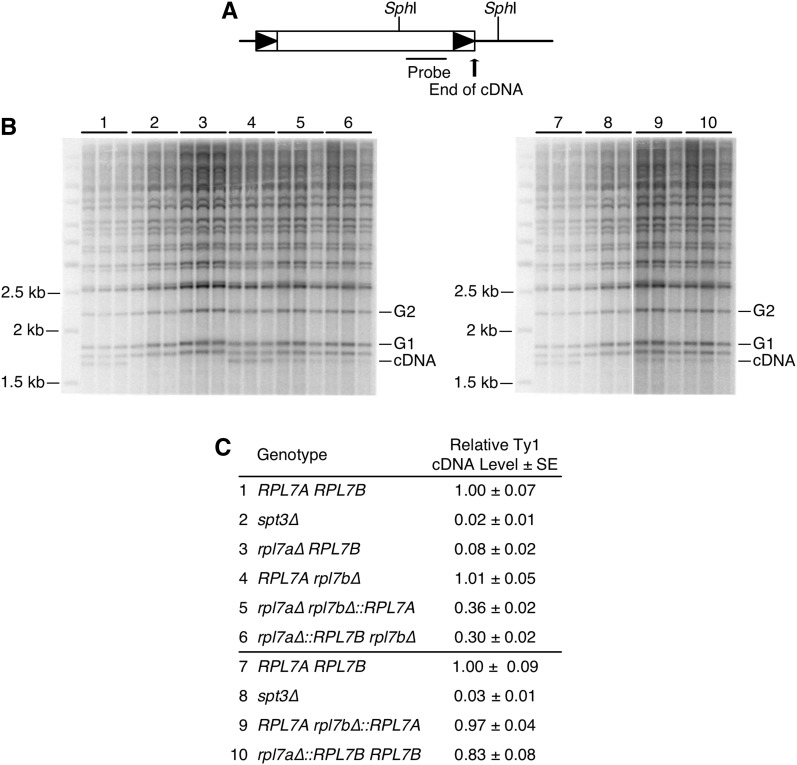
Rpl7 and snR39/59 isoforms expressed affect Ty1 cDNA accumulation in a dosage-and isoform-dependent manner. (A) Schematic of a chromosomal Ty1 element, with the positions of *Sph*I sites in Ty1 and flanking genomic DNA indicated. The position of a riboprobe used in Southern blot analysis is indicated. (B) Southern blot analyses of three biological replicates of genomic DNA from each strain. Numbers correspond to strain genotypes indicated below in (C). The amount of unintegrated Ty1 cDNA is measured relative to genomic Ty1 elements in *Sph*I-digested total DNA. The size of each Ty1:genomic DNA junction band is determined by the distance between the internal Ty1 *Sph*I site and the nearest *Sph*I site in flanking genomic DNA. The 1.7 kb Ty1 cDNA band is the fastest-migrating band because of the lack of flanking genomic DNA. The relative Ty1 cDNA level is the average ratio of the ^32^P-signal in the unintegrated Ty1 cDNA band (cDNA) relative to the average of two different Ty1:genomic DNA junction bands (G1 and G2) in three samples. Unmarked lanes: molecular weight marker. (C) Average (±SE) Ty1 cDNA level in each mutant relative to the *RPL7A RPL7B* strain. The *spt3∆* strain, in which Ty1 RNA and cDNA are markedly decreased, was analyzed as a negative control.

Given that Ty1 retrotransposes efficiently, but retrosomes rarely form in a mutant with modestly reduced Rpl7 and 60S subunit levels, we determined whether Ty1 cDNA accumulation, a step in retrotransposition between retrosome formation and transposition into the genome, is regulated by *RPL7* paralogs. Unintegrated Ty1 cDNA levels in paralog deletion strains were measured relative to genomic copies of Ty1 via quantitative Southern blot analysis (Scholes *et al.* 2001). Deletion of *RPL7A* decreased Ty1 cDNA levels to 8% of the wild-type strain, whereas deletion of *RPL7B* had no effect on cDNA accumulation, demonstrating that Ty1 cDNA accumulation is a paralog-specific function of *RPL7A* (Figure 8). When the *RPL7B* CRI was substituted for the *RPL7A* CRI at the *RPL7A* locus, cDNA levels were reduced to 30% of that in the wild-type strain. On the other hand, substituting *RPL7A* for the *RPL7B* CRI at the *RPL7B* locus increased cDNA levels from 8 to 36% of that in the wild-type strain. Equivalent levels of Ty1 cDNA in the *rpl7a∆ rpl7b∆*::*RPL7A* and *rpl7a∆*::*RPL7Brpl7b∆* mutants was not predicted by the substantially different Rpl7 and 60S ribosomal subunits levels between these mutants. Higher cDNA levels resulting from expression of Rpl7a/snR39, rather than Rpl7b/snR59 from both the *RPL7A* and *RPL7B* loci, suggested that Rpl7a/snR39 isoforms could have a specialized role in Ty1 cDNA accumulation. To determine whether this function is unique to Rpl7a/snR39, or can be performed by Rpl7b/snR59 at a higher dosage, cDNA levels in homogenic strains were measured (Figure 8). Expression of the *RPL7B* CRI from both loci increased the relative level of cDNA from 30 to 83% compared to expression from the *RPL7A* locus only. Thus, a mutant expressing Rpl7b/snR59 with a polysome profile that is comparable to the wild-type strain has efficient cDNA accumulation, indicating that cDNA accumulation is not a distinct function of Rpl7a/snR39. Nonetheless, equivalent levels of Ty1 cDNA in a mutant with low levels of Rpl7a, and a severe 60S ribosomal subunit defect, as well as a mutant with higher levels of Rpl7b, and a mild 60S subunit defect, suggests that Rpl7a or snR39 is more active than Rpl7b or snR59 at some aspect of Ty1 cDNA synthesis or stability.

### Unaltered mechanism of cDNA integration in the rpl7a∆::RPL7B rpl7b∆ mutant

The rate of Ty1 retromobility in the *rpl7a∆*::*RPL7Brpl7b∆* mutant was similar to that of the *RPL7ARPL7B* strain (Table 1), despite a low levels of Ty1 retrosomes, and a 70% reduction in unintegrated Ty1 cDNA. Therefore, we determined whether Ty1 cDNA is introduced into the genome of the *rpl7a∆*::*RPL7Brpl7b∆* mutant via a mechanism that is different than that of the wild-type strain, which might uncover evidence for a specialized function Rpl7b/snR59 in retromobility. Ty1 cDNA integration is normally much more efficient than recombination with genomic Ty1 sequences. Accordingly, the homologous recombination factor, Rad52, is dispensable for retromobility, and deletion of *RAD52* increases Ty1 retromobility by stimulating cDNA synthesis (Table 2) (Sharon *et al.* 1994; Rattray *et al.* 2000; Curcio *et al.* 2007). Deletion of *RAD52* also increased retromobility in the *rpl7a∆*::*RPL7Brpl7b∆* strain, suggesting that cDNA recombination is rare and integration remains the major mechanism of retromobility (Table 2). A second possibility is that the target site specificity of cDNA integration is altered when only Rpl7b/snR59 is expressed. Therefore, independent Ty1*HIS3* integration sites in the *rpl7a∆*::*RPL7Brpl7b∆* mutant were compared to those in a wild-type strain. Following selection for His^+^ prototrophs, the location of Ty1*HIS3* was determined by sequencing Ty1*HIS3* cDNA:genomic DNA junctions amplified by TAIL PCR (Table S3). Integration is usually targeted to a ∼750-bp window upstream of Pol III transcribed genes (Ji *et al.* 1993; Devine and Boeke 1996; Baller *et al.* 2012; Mularoni *et al.* 2012). In the *RPL7ARPL7B* strain, 12 of 17 Ty1*HIS3* retromobility events integrated within 475 bp upstream of a Pol III-transcribed gene, and one was 150 bp downstream of a Pol III-transcribed gene. In the remaining four isolates, Ty1*HIS3* cDNA was present within a tandem array of Ty1 cDNAs, so genomic sequences flanking these arrays could not be identified by TAIL PCR. A similar integration profile was observed for 15 independent Ty1*HIS3* cDNA integration events in the *rpl7a∆*::*RPL7Brpl7b∆* mutant. The two strains had equivalent fractions of Ty1*HIS3* elements integrated upstream of Pol III-transcribed genes (11/15 *vs.* 12/17) and within multimeric arrays (4/15 *vs.* 4/17). One Ty1*HIS3* element integrated into an ORF >6 kb away from the nearest Pol III-transcribed gene, but this low fraction of ORF disruption is not inconsistent with that observed in a study of >150,000 Ty1*HIS3* insertions in a wild-type strain (Baller *et al.* 2012). We conclude that Ty1 target specificity is generally preserved in the *rpl7a∆*::*RPL7Brpl7b∆* mutant, and, therefore, that expression of Rpl7b/snR59 alone does not result in an altered mechanism of Ty1 retromobility.

**Table 2:** Effect of *RAD52* disruption on Ty1*his3AI* retromobility

	Frequency of Ty1*his3AI* Retromobility*^a^* ± SE × 10^−7^		
Genotype	*RAD52*	*rad52*	*rad52/RAD52* Ratio
*RPL7A RPL7B*	3.78 ± 0.13	102.63 ± 23.55	27
*RPL7A rpl7b∆*	3.41 ± 0.48	88.10 ± 11.21	26
*rpl7a∆*::*RPL7B rpl7b∆*	3.63 ± 0.74	57.30 ± 4.18	16

a The mean of the number of His^+^ prototrophs per culture divided by the total number of cells plated.

## Discussion

### Different Rpl7 or snoRNA levels underlie paralog-specific functions of RPL7A and RPL7B

The question of whether the diverse phenotypic consequences of deleting one paralog or the other of an RP gene pair can be explained by differences in RP gene dosage, or constitute evidence of “specialized” ribosomes containing distinct subsets of RP isoforms has been widely debated (Komili *et al.* 2007; Parenteau *et al.* 2011, 2015; Steffen *et al.* 2012; Xue and Barna 2012; Slavov *et al.* 2015; Dinman 2016). Based on the mislocalization of Rpl7b, but not Rpl7a, when certain ribosome biogenesis factors were depleted, it was proposed that Rpl7 isoforms are incorporated into heterogenous ribosomes that selectively translate specific mRNAs, and give rise to paralog-specific functions of *RPL7A* and *RPL7B* (Mauro and Edelman 2002, 2007; Komili *et al.* 2007; Xue and Barna 2012). However, phenotypic comparisons of strains expressing either Rpl7a/snR39 or Rpl7b/snR59 from the robust *RPL7A* locus, or weaker *RPL7B* locus, suggest that paralog-specific functions of *RPL7A* and *RPL7B* can be explained by differences in the level of Rpl7 or *RPL7*-encoded snoRNA expression, and the effect of depleting these gene products on 60S ribosomal subunit levels. For example, the efficiencies of *ASH1* mRNA localization, and Ty1 retromobility in mutants expressing either *RPL7* CRI from the *RPL7A* locus, were similar to each other, and to the wild-type strain, whereas mutants with either *RPL7* CRI at the *RPL7B* locus had marked defects in these processes. Because the *rpl7a∆ RPL7B* and *rpl7a∆ rpl7b∆*::*RPL7A* mutants have severe reductions in 60S ribosomal subunits that lead to halfmer ribosomes, and the *rpl7a∆*::*RPL7Brpl7b∆* and *RPL7Arpl7b∆* mutants have minor reductions in 60S ribosomal subunits; these observations indicate that *ASH1* mRNA localization and Ty1 retromobility are correlated with the dosage of Rpl7 and snR39/59, and not with the specific isoforms expressed. The Ty1 retromobility phenotypes of strains expressing an epitope-tagged Rpl7b, the level of which increased when *RPL7A* is deleted, support this view, and further suggest that only the dosage of single Rpl7 isoform need be altered to produce different phenotypic outcomes. Dosage of the encoded product(s) also explains the paralog-specific regulation of Ty1 retromobility by *RPL31A*. Together, these findings support the hypothesis that phenotypic variation in complex cellular processes can result from different degrees of ribosomal subunit imbalance.

Other phenotypes examined here differed depending on which CRI was expressed from a particular locus. There was greater resistance to tunicamycin when Rpl7b/snR59 *vs.* Rpl7a/snR39 was expressed from the *RPL7B* locus, and Ty1 RNA localization was significantly more robust when Rpl7a/snR39 was expressed from the *RPL7A* locus. These phenotypic differences do not necessarily result from distinct, or even specialized, functions of the Rpl7 or snoRNA isoforms, because Rpl7a is more highly expressed from either locus than Rpl7b. The reason for this expression difference was not investigated, but it likely reflects the negative regulatory effects that *RPL7B* introns have on *RPL7B* mRNA expression (Parenteau *et al.* 2011). Growth rates and polysome profiles indicated that mutants expressing Rpl7a/snR39 have a less severe deficiency in 60S ribosomal subunits that parallels their higher Rpl7 levels. Thus, a different extent of 60S subunit depletion is the likely explanation for the phenotypic differences associated with disparate *RPL7* CRI at the same locus. The importance of Rpl7 and snoRNA dosage and irrelevance of isoform in these phenotypes was further illustrated by the effects of increasing the dosage of Rpl7b/snR59. Tunicamycin resistance was suppressed by expressing Rpl7b/snR59 from the *RPL7A* locus, and the Ty1 RNA localization defect was rescued by expressing Rpl7b/snR59 from both *RPL7* loci. The dependence of the phenotypes above on the dosage of Rpl7 or snoRNA strongly suggests that neither Rpl7 nor snR39/59 isoforms have distinct functions in the relevant cellular processes.

Ty1 cDNA accumulation was the single phenotype studied here that was not strictly correlated with the extent of 60S ribosomal subunit depletion. Similar levels of Ty1 cDNA in a *rpl7a∆ rpl7b∆*::*RPL7A* mutant, which has a severe 60S ribosomal subunit biogenesis defect, and an *rpl7a∆*::*RPL7Brpl7b∆* mutant, which has only a modest reduction in 60S ribosomal subunits, suggests that Rpl7a or snR39 could have a specialized role in promoting cDNA synthesis or stability. Nonetheless, this is not a unique role, since expressing Rpl7b/snR59 from both *RPL7* loci rescued the defect in cDNA accumulation in mutants expressing Rpl7b/snR59 from either single locus. The finding that defective cDNA accumulation is correlated with a depletion of Rpl7a/snR39 or Rpl7b/snR59, albeit to substantially different levels depending on the isoforms, argues against the idea that control of cDNA accumulation is an extraribosomal function of Rpl7 isoforms. Rather, these observations are consistent with Rpl7a being more active than Rpl7b in a specific ribosome-associated function. Numerous host cofactors and signaling pathways influence Ty1 cDNA levels (Curcio *et al.* 2015), and, thus, the translation of an mRNA that encodes a Ty1 cofactor could be more efficient in a strain expressing Rpl7a/snR39. The N-terminal domain of Rpl7, which contains four of the five amino acid substitutions that differentiate the Rpl7 isoforms and forms an α-helix located on the surface of the ribosome (Wilson and Doudna Cate 2012), could differentially recruit a factor that influences the efficiency of translation of specific mRNAs. Three of the four N-terminal substitutions in Rpl7a are serine and threonine residues, suggesting the possible involvement of signaling via post-translational modification of Rpl7a.

Although evidence for neofunctionalization of Rpl7, snR39/59 or Rpl31 isoforms was not uncovered, it remains possible that these isoforms have distinct, dosage-independent functions in other processes not tested here, and that *RPL7* and *RPL31* paralogs are differentially expressed as environmental conditions vary (Parenteau *et al.* 2011, 2015). The chimeragenic and homogenic *RPL7* strains described here provide a useful tool for testing the isoform specificity of other cellular processes under a variety of environmental conditions. It is also possible that isoforms encoded by RP gene pairs other than *RPL7* paralogs have unique functions in specialized ribosomes. Nonetheless, this study highlights the importance of considering the relative levels of each paralog’s protein products in the absence of the corresponding paralog when assigning RP isoform-specific functions. Other studies have used the rate of cell growth and polysome profiling in paralog-deletion strains as informative proxies of relative RP levels that are incorporated into functional ribosomes (Steffen *et al.* 2008, 2012), and these findings also support the idea that RP dosage and levels of functional ribosomes underlie many complex cellular phenotypes in yeast.

### The effects of Rpl7 depletion on intermediate steps in Ty1 retromobility

Uncovering the mechanism by which Rpl7 or the *RPL7*-encoded snoRNAs regulate Ty1 retromobility was not the primary goal of this study, but several mechanistic conclusions can be drawn from our findings. First, Ty1 mRNA is not likely to be translated preferentially on ribosomes containing Rpl7a or snR39-modified rRNA, since equivalent levels of Gag accumulate when either the *RPL7* CRI was expressed from the *RPL7A* locus. Second, depletion of Rpl7, even to the point of a severe deficiency in 60S ribosomal subunits, caused a <2-fold decrease in the level of Gag, and overexpressing Ty1 proteins only weakly rescued the Ty1 retromobility defect of an *rpl7a∆ RPL7B* mutant; therefore, reduced Gag is not likely to be the major cause of the decrease in retromobility in *rpl7a∆* mutants. Many RP genes influence Ty1 retromobility (Harger *et al.* 2001; Griffith *et al.* 2003; Dakshinamurthy *et al.* 2010; Risler *et al.* 2012; Suresh *et al.* 2015), and several others that are superfluous for Ty1 Gag accumulation have been characterized (Suresh *et al.* 2015). Third, Ty1 retromobility was more sensitive to Rpl7 or snR39/59 depletion than Gag accumulation, suggesting that Rpl7 or snR39/59 might regulate retrotransposition at a post-translational level. However, it is important to note that our studies have not addressed the effect of Rpl7 depletion on translational frameshifting from the *GAG* to *POL* ORF, the efficiency of which impacts the level of Ty1 retrotransposition (Curcio *et al.* 2015). Fourth, localization of Ty1 RNA to retrosomes, and cDNA accumulation, had very low thresholds for Rpl7 and snR39/59 levels, suggesting that Rpl7 or *RPL7*-encoded snoRNAs might directly influence Ty1 RNA localization. While retrotransposition was efficient even in a mutant with reduced retrosomes and cDNA, the findings suggest a model in which Rpl7 or snR39/59 control the cotranslational localization of Ty1 RNA in retrosomes. Rpl7 or ribosomal RNA modifications made by snR39/59 could facilitate the interaction between Gag and Ty1 RNA on translating ribosomes, which is critical for retrosome formation (Doh *et al.* 2014). Another possibility supported by our findings is that depletion of Rpl7, like that of a few other 60S ribosomal subunit proteins, increases expression of a truncated form of Gag (p22-Gag) that potently inhibits retromobility and reduces retrosome formation (Saha *et al.* 2015; Suresh *et al.* 2015). It is not obvious how a shortage of 60S ribosomal subunits would increase the expression of the internal Ty1 transcript that encodes p22-Gag, but it could be via an indirect effect on cell metabolism or the cell cycle.

### Variable phenotypic consequences of reduced expression of Rpl7

The results presented here suggest that different cellular pathways have varied thresholds for 60S ribosomal subunit depletion. For example, the dosage of Rpl7b/snR59 produced from the *RPL7A* locus, which results in a moderately reduced level of 60S ribosomal subunits, and slower growth relative to the wild-type strain, is sufficient for wild-type levels of *ASH1* mRNA localization, and Ty1 retromobility, but insufficient for Ty1 RNA localization. Depletion of other 60S ribosomal subunits by deletion of different RP gene paralogs, or 60S subunit biogenesis factors, also quantitatively alters distinct cellular processes, including replicative life span and translation of the L-A virus mRNA (Ohtake and Wickner 1995; Steffen *et al.* 2008). Therefore, phenotypic variation in many cellular processes might be linked to varying degrees of ribosomal subunit imbalance. The asymmetric regulation and expression of many RP gene pairs also supports the idea that differences in ribosome levels could underlie paralog-specific phenotypes (Parenteau *et al.* 2011, 2015).

In humans, mutations in RP genes, most of which are present in a single copy, have been linked to a plethora of inherited developmental abnormalities, and somatic mutations in RP genes have been found in specific cancers. Tissue-specific defects that characterize specific mutations in various RP genes may reflect unique functions of ribosomes lacking a particular RP, or imbalances in ribosomal subunits that affect some cellular pathways more strongly than others, and each of these cellular pathways may be more or less critical for differentiation in specific cell types (Xue and Barna 2012; McCann and Baserga 2013; De Keersmaecker *et al.* 2015). In mice, ribosomopathy-associated tissue-specific abnormalities are, in some cases, linked to impaired translation of specific subsets of mRNAs (Xue and Barna 2012; McCann and Baserga 2013). For example, depletion of Rpl38 in certain tissues blocks translation of the mRNAs of a specific subset of homeobox genes during mouse embryogenesis; these mRNAs have specific *cis*-acting regulatory sequences in their 5′ UTRs that govern their dependence on Rpl38 (Kondrashov *et al.* 2011; Xue *et al.* 2015). What remains to be determined in human ribosomopathies and mouse models is whether the depletion of a specific RP changes the composition or level of functional ribosomes (McCann and Baserga 2013). Because Rpl7 is required for an early step in ribosome biogenesis in yeast, and there is a tight correlation between Rpl7 and 60S subunit levels (Jakovljevic *et al.* 2012), it is likely that the phenotypes associated with Rpl7 and snoRNA depletion in yeast are a result of a reduced level of functional ribosomes, rather than an increased percentage of ribosomes lacking Rpl7. Thus, this study supports a model in which subtle differences in ribosomal subunit biogenesis caused by depletion of a single RP result in varied phenotypic consequences, perhaps because translation of specific subsets of mRNAs have different sensitivities to imbalances between ribosomal subunits (Ohtake and Wickner 1995; Jakovljevic *et al.* 2012; Steffen *et al.* 2012). Characterization of global translation patterns in yeast strains that are homogenic or chimeragenic for a single RP paralog, or harbor a single native allele, could provide a systematic means of identifying classes of mRNAs that are differentially sensitive to ribosomal subunit imbalances.

## Supplementary Material

Supplemental material is available online at www.g3journal.org/lookup/suppl/doi:10.1534/g3.116.035931/-/DC1.
