# Supplementary figures and images for: Paralog-Specific Functions of RPL7A and RPL7B Mediated by Ribosomal Protein or snoRNA Dosage in Saccharomyces cerevisiae
Source: G3 (Bethesda). 2016 Dec 19;7(2):591–606. doi: 10.1534/g3.116.035931 (PMC5295604; doi:10.1534/g3.116.035931)

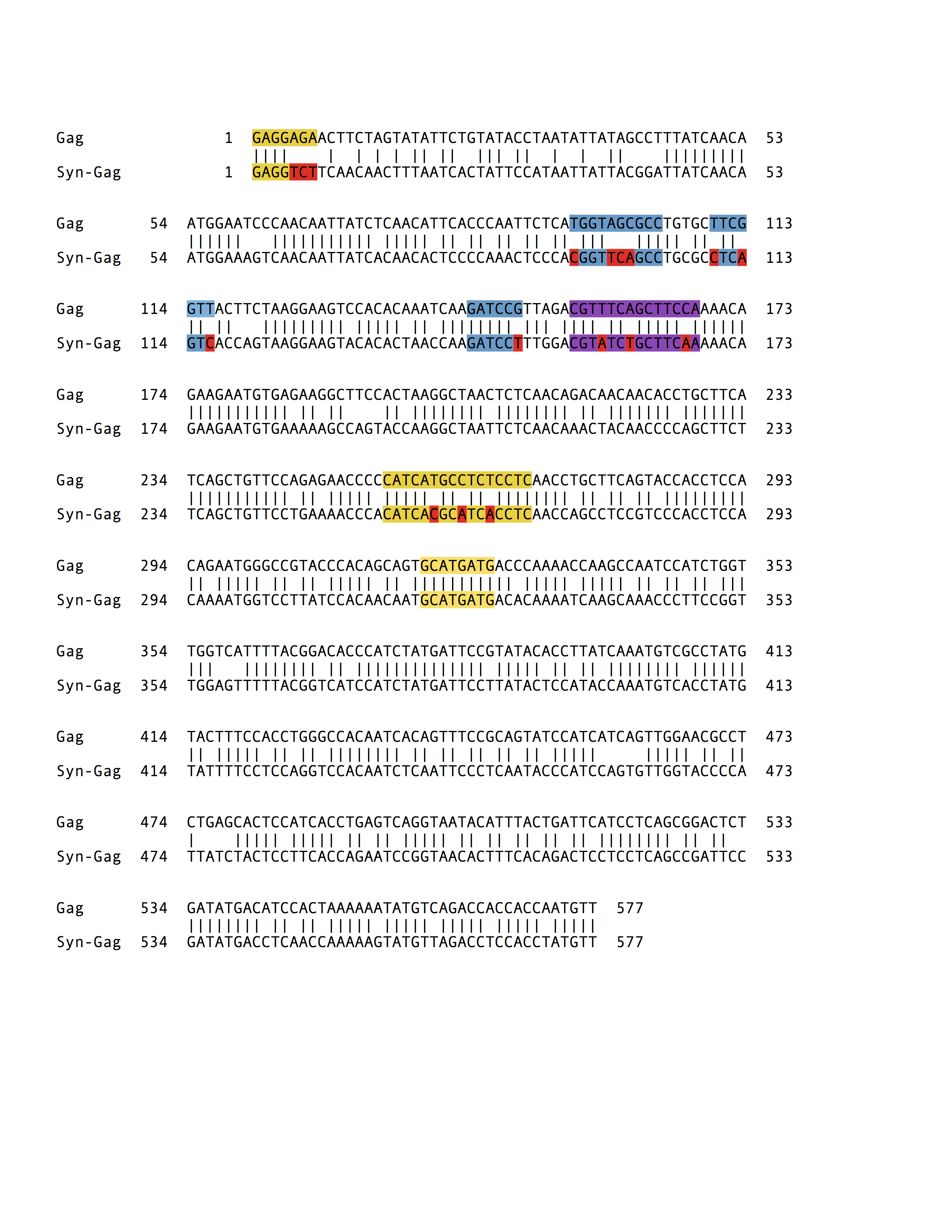

Supplement: Supplementary file 1 [file 591FigureS1.tif]
