# Supplementary material for: Paralog-Specific Functions of RPL7A and RPL7B Mediated by Ribosomal Protein or snoRNA Dosage in Saccharomyces cerevisiae
Source: G3 (Bethesda). 2016 Dec 19;7(2):591–606. doi: 10.1534/g3.116.035931 (PMC5295604; doi:10.1534/g3.116.035931)
Supplement: Supplementary file 2 [file 591TableS1.docx]

| **S1 Table. Yeast strains used in this study** | | |
| --- | --- | --- |
| Strain name | Genotype | Source |
| BY4741 | *MAT*a *his3∆1 leu2∆0 met15∆0 ura3∆0* | Open Biosystems |
| JC3212 | *MAT*a *his3∆1 leu2∆0 met15∆0 ura3∆0 Ty1his3AI[∆1]-3114* | Mou et al. 2006 |
| JC3807 | *MAT*a *his3∆1 leu2∆0 met15∆0 ura3∆0 Ty1his3AI[∆1]-3114 Ty1:GFP-3566* | Scholes et al. 2003 |
| JC5212 | *rpl7b∆::kanMX4* derivative of JC3212 | This study |
| JC5224 | *rpl7a∆::kanMX4* derivative of JC3212 | This study |
| JC5101 | *tif4631∆::kanMX4* derivative of JC3212 | This study |
| JC5268  JC5269 | *rpl7a∆::kanMX4* derivative of JC3807 | This study |
| JC5319  JC5320 | *rpl7b∆::kanMX4* derivative of JC3807 | This study |
| JC5896 JC5897 JC5898 | *rpl7a∆::GAL1_P_:I-SceI-hyg-K.l.URA3* derivative of JC3212 | This study |
| JC5899 JC5900 JC5901 | *rpl7b∆::GAL1_P_-I-SceI-hyg-K.l.URA3* derivative of JC3212 | This study |
| JC5902 JC5903 | *rpl7b∆::RPL7A* derivative of JC5900 | This study |
| JC5904 | *rpl7b∆::RPL7A* derivative of JC5901 | This study |
| JC5905 JC5906 | *rpl7a∆::RPL7B* derivative of JC5896 | This study |
| JC5907 | *rpl7a∆::RPL7B* derivative of JC5898 |  |
| JC5910 | *rpl7b∆::kanMX4* derivative of JC5905 | This study |
| JC5911 | *rpl7b∆::kanMX4* derivative of JC5906 | This study |
| JC5912 | *rpl7b∆::kanMX4* derivative of JC5907 | This study |
| JC5913 | *rpl7a∆::kanMX4* derivative of JC5902 | This study |
| JC5914 | *rpl7a∆::kanMX4* derivative of JC5903 | This study |
| JC5915 | *rpl7a∆::kanMX4* derivative of JC5904 | This study |
| JC6221 JC6222 JC6223 | *rad52::hisG-URA3-hisG* derivative of JC3212 | This study |
| JC6224 JC6225 JC6226 | *rad52::hisG-URA3-hisG* derivative of JC5212 | This study |
| JC6227 JC6228 JC6229 | *rad52::hisG-URA3-hisG* derivative of JC5912 | This study |
| JC3709 | *MAT*a *his3∆1 leu2∆0 met15∆0 ura3∆0 trp1::hisG Ty1his3AI[∆1]-3114* | This study |
| JC5291 | *RPL7B:9xMyc-K.l.TRP1* derivative of JC3709 | This study |
| JC5293 | *rpl7a∆::kanMX4 RPL7B:9xMyc-K.l.TRP1* derivative of JC3709 | This study |
| JC5295 | *RPL7A:9xMyc-K.l.TRP1* derivative of JC3709 | This study |
| JC5297 | *RPL7A:9xMyc-K.l.TRP1 rpl7b∆::kanMX4* derivative of JC3709 | This study |
| JC6129  JC6130  JC6131 | *rpl31a∆::GAL1-I-SceI-hygB-K.l.URA3* derivatives of JC3212 | This study |
| JC6132  JC6133 | *rpl31a∆::RPL31B* derivatives of JC6130 | This study |
| JC6134 | *rpl31a∆::RPL31B* derivatives of JC6131 | This study |
| JC6135 | *rpl31b∆::kanMX4* derivative of JC6132 | This study |
| JC6136 | *rpl31b∆::kanMX4* derivative of JC6133 | This study |
| JC6137 | *rpl31b∆::kanMX4* derivative of JC6134 | This study |
