# Supplementary material for: Paralog-Specific Functions of RPL7A and RPL7B Mediated by Ribosomal Protein or snoRNA Dosage in Saccharomyces cerevisiae
Source: G3 (Bethesda). 2016 Dec 19;7(2):591–606. doi: 10.1534/g3.116.035931 (PMC5295604; doi:10.1534/g3.116.035931)
Supplement: Supplementary file 3 [file 591TableS2.docx]

**Table S2. Oligonucleotide Primers Used In This Study**

| Primer | Sequence (5’-3’) |
| --- | --- |
| H3HOPA2 | TCT CCT ACT TTC TCC CTT TGC AAA CC |
| HISOUT3 | CTT CGT TTA TCT TGC CTG CTC |
| LTR-PROBE | GTG GAA GCT GAA ACG CAA GGA TTG AT |
| TRPHOP-SE | AGA TTG TAC TGA GAG TGC ACC |
| TY5253A | GGA CAG ATT CAC TTA TCG CGT GT |
| TYAOUT2 | TCT CTG GAA CAG CTG ATG AAG |
| PJ4 | ACC ACT TGC GCT TAT TTC TTG GAA GTG TTG TAT CTC AAA ATG AGA CTG TGC GGT ATT TCA CAC CG |
| PJ5 | ATG TTA TTA TAA ACT ACT TAC CAA AAA AAG TGT TGT ATT ACG GGC AGA TTG TAC TGA GAG TGC AC |
| PJ143 | GAT CTA CCA CCG CTC TGG AAA GTG |
| PJ371 | TGTGTGCAACGGATGGATGGTAG |
| PJ372 | TCGTAAACGAAGACTGGCGAAGC |
| PJ457 | ACT GCC GAT TCT GGC ATT CC |
| PJ458 | CAG CGA CTT GTT CAG CAG TC |
| PJ459 | GCG ACT TCT GAG AAT CAT TGC CA |
| PJ460 | GTG CAT AGA AGC GTT CGA AG |
| PJ461 | CTG AGT TCC GAC TAC TAC TCC A |
| PJ462 | TGG CAT CAC GCT TAG CTT GA |
| PJ463 | ACA GTT GAG GTA CCC ACA CG |
| PJ464 | TCT CTA GCT GTA CGT GAG TG |
| PJ471 | GTGCTCACAATTGGATTGGCA |
| PJ472 | CGCCTCAGTGGTTGAGAGAC |
| PJ564 | AGG ACT TAG ATA AAA TAA GTA GTT ATA TGT AAT GTG ACT TCA ACC AGT CCT CGA GGC CAG AAG AC |
| PJ565 | TTC GGT AAC CGT GAA GAA TTC ATC AAC AAA TTG GTT AAG TCC ATG AAC TCC GGT TCT GCT GCT AG |
| PJ566 | AGA AAT ATT ATT TAT TAT ACA TTG AGA TTA TTG CCA CAC GGA ATA ATG CCT CGA GGC CAG AAG AC |
| PJ567 | TTC GGT AAC CGT GAA GAA TTC ATC AAT AAA TTG GTT AAG GCT ATG AAC TCC GGT TCT GCT GCT AG |
| PJ748 | GCT TCG TAT GGC AAC CAA CC |
| PJ750 | TTC GCG AAG TAA CCC TTC GTG GA |
| PJ751 | GTA AAA CGG TTC ATC CTT ATG CAG |
| PJ757 | ATA AAT CCA AAT AAC CAA CCA AGC AAA TTA AGA TCA CAA TGT CCA CTG AGT ATG TCA AAA ACT AC |
| PJ758 | CTC AAT CTT GCA GAT TCT TGG C |
| PJ759 | AAA ATA AGT AGT TAT ATG TAA TGT GAC TTC AAC CAG TTT AGT TCA TAG CCT TAA CCA ATT TAT TG |
| PJ796 | TAT AGC CTT TAT CAA CAC TGG AAT CCC AAC AAT TAT CTAA |
| PJ813 | GGT TCA TAT AAA TCC AAA TAA CCA ACC AAG CAA ATT AAG ATC ACA TAG GGA TAA CAG GGT AAT CCG CGC GTT GGC CGA TTC AT |
| PJ814 | AGG ACT TAG ATA AAA TAA GTA GTT ATA TGT AAT GTG ACT TCA ACC AGT TTC GTA CGC TGC AGG TCG AC |
| PJ842 | AGT AAG TAG ACC CAT ATT ACA AAT CTC CAT CAA CGT CAT ATA GGG ATA ACA GGG TAA TCC GCG CGT TGG CCG ATT CAT |
| PJ843 | TAT TTA TTA TAC ATT GAG ATT ATT GCC ACA CGG AAT AAT GTT CGT ACG CTG CAG GTC GAC |
| PJ845 | AGT AAG TAG ACC CAT ATT ACA AAT CTC CAT CAA CGT CAT AAT GGC CGC TGA GTA TGT ATA CG |
| PJ846 | TAT TTA TTA TAC ATT GAG ATT ATT GCC ACA CGG AAT AAT GTT AGT TCA TGG ACT TAA CC |
| PJ893 | GAA AGA ATT AAT AAG CAA ACA GAA CTC AAT CAA AGG AAA ATA GGG ATA ACA GGG TAA TCC GCG CGT TGG CCG ATT CAT |
| PJ894 | TTT ATT AAT ATA AAA TGA GTA TTA TTT TTA TTT AAT TGA TTT CGT ACG CTG CAG GTC GAC |
| PJ895 | GAA AGA ATT AAT AAG CAA ACA GAA CTC AAT CAA AGG AAA AAT GGC CGG TTT AAA AGA CG |
| PJ896 | TTT ATT AAT ATA AAA TGA GTA TTA TTT TTA TTT AAT TGA TTT AAG CAT CTT CTT CAA CGA CAA CGG |
| PJ897 | CTA AGA TAA TGG GGC TGT TT |
| PJ934 | TTC CCA CGT AAA GAC AAC GC |
| PJ935 | CGT GAA CTC ACA TAT ATC CC |
| PJ944 | TAA TGC GGC CGC TTA CCC TGT TAT CCC TAG CG |
| PJ948 | GGG GGA TCC AGC GTT CGA AGA TTG ATC CG |
| PJ964 | GGG GGA AGC TTA TGA TAC TGC CGA TTC TGG C |
| PJ970 | GTA TAT CCG TGA TCG AAC CG |
| PJ1169 | TAA TGC GGC CGC ACT TCT AAA TAA GCG AAT TTC |
| PJ1220 | GGG AAC TGC TGA TCA TCT C |
| PJ1221 | GGG GGC TCG AGT AAG CGG TTT GCG ATT CAT GGC |
| PJ1227 | TGA AAC ACC ATG GAG TAG GC |
| PJ1228 | GAG ACA GTA GAG AAT TGA TAC ATG G |
| PJ1229 | ATC AGG ATG ACC AAT CTA TTG CGC AGG |
| PJ1230 | CTT TCC TCC AAT TAC CTT TCC |
| PJ1285 | GAT CGA CTC GAG ATG TCA AGC TTA TAC ATC |
| PJ1286 | GAT CGA GGA TCC TCA ATT CTC TAC TGT CTC AG |
| PJ1347 | GGG GGG GGG GAT CCC CAT GTC GAA AGC TAC ATA TAA GG |
| PJ1348 | GGG GGG GGG GCG CGC CG GGT AAT AAC TGA TAT AAT TAA ATT GAA GCT C |
