# Supplementary material for: Paralog-Specific Functions of RPL7A and RPL7B Mediated by Ribosomal Protein or snoRNA Dosage in Saccharomyces cerevisiae
Source: G3 (Bethesda). 2016 Dec 19;7(2):591–606. doi: 10.1534/g3.116.035931 (PMC5295604; doi:10.1534/g3.116.035931)
Supplement: Supplementary file 4 [file 591TableS3.docx]

**Table S3. Genomic location of Ty1*HIS3* retrotransposition events**

| **Strain genotype** | | **Chr** | | **Orientation** | | **Coordinate** | | **Nearest**  **Pol III gene** | | **Distance from Pol III gene (bp)** | | | **Details** | |  |  |
| --- | --- | --- | --- | --- | --- | --- | --- | --- | --- | --- | --- | --- | --- | --- | --- | --- |
| *RPL7A RPL7B* | | V | | Watson | | 441869 | | *SCR1* | | 118 | |  | | | | |
|  | | VII | | Watson | | 74191 | | *tV(AAC)G3* | | 289 | |  | | | |  |
|  | | VII | | Watson | | 701139 | | *tK(UUU)G2* | | 91 | |  | | | |  |
|  | | IX | | Watson | | 324774 | | *tT(AGU)I2* | | 474 | |  | | | |  |
|  | | X | | Watson | | 59356 | | *tT(AGU)J* | | 184 | |  | | | |  |
|  | | XII | | Crick | | 488998 | | *RDN5-6* | | 351 | | In uncharacterized ORF *YLR161W* | | | |  |
|  | | XII | | Crick | | 796709 | | *tE(UUC)L* | | 469 | |  | | | |  |
|  | | XII | | Watson | | 468688 | | *RDN5-2* | | 125 | |  | | | |  |
|  | | XII | | Crick | | 168191 | | *tS(AGA)L* | | 166 | |  | | | |  |
|  | | XIV | | Watson | | 632228 | | *tP(AGG)N* | | 311 | |  | | | |  |
|  | | XV | | Watson | | 110812 | | *tG(UCC)O* | | 150 | |  | | | |  |
|  | | XV | | Watson | | 227061 | | *tG(GCC)O1* | | 380 | |  | | | |  |
|  | | XVI | | Watson | | 210539 | | *tE(UUC)P* | | 276 | |  | | | |  |
|  | | NA | | NA | | NA | | NA | | NA | | In multimeric Ty1 array | | | |  |
|  | | NA | | NA | | NA | | *NA* | | NA | | In multimeric Ty1 array | | | |  |
|  | | NA | | NA | | NA | | *NA* | | NA | | In multimeric Ty1 array | | | |  |
|  | | NA | | NA | | NA | | *NA* | | NA | | In multimeric Ty1 array | | | |  |
| *rpl7a∆::RPL7B rpl7b∆* | | IV | | Watson | | 1175595 | | *tM(CAU)D* | | 234 | |  | | | |  |
|  | | VIII | | Watson | | 116540 | | *tT(AGU)H* | | 361 | |  | | | |  |
|  | | VIII | | Watson | | 85511 | | tV(AAC)H | | 140 | |  | | | |  |
|  | | XI | | Crick | | 84121 | | *tL(UAA)K* | | 87 | |  | | | |  |
|  | | XII | | Crick | | 963467 | | *tL(UAA)L* | | 412 | |  | | | |  |
|  | | XII | | Watson | | 468583 | | *RDN5-2* | | 230 | |  | | | |  |
|  | | XII | | Watson | | 485593 | | *RDN5-5* | | 104 | | In uncharacterized ORF *YLR159W* | | | |  |
|  | | XII | | Watson | | 459551 | | *RDN5-1* | | 125 | |  | | | |  |
|  | | XIII | | Watson | | 815069 | | *tQ(CUG)M* | | 6752 | | In *RCE1* ORF | | | |  |
|  | | XIV | | Watson | | 632398 | | *tN(GUU)N2* | | 201 | |  | | | |  |
|  | | XV | | Watson | | 438387 | | *tK(UUU)O* | | 256 | |  | | | |  |
|  | | NA | | NA | | NA | | *NA* | | NA | | In multimeric Ty1 array | | | |  |
|  | | NA | | NA | | NA | | *NA* | | NA | | In multimeric Ty1 array | | | |  |
|  | | NA | | NA | | NA | | NA | | NA | | In multimeric Ty1 array | | | |  |
|  | | NA | | NA | | NA | | NA | | NA | | In multimeric Ty1 array | | | |  |
|  |  | |  | |  | |  | |  | |  | | |  |  |  |
|  |  | |  | |  | |  | |  | |  | | |  |  |  |
|  |  | |  | |  | |  | |  | |  | | |  |  |  |
|  |  | |  | |  | |  | |  | |  | | |  |  |  |
