# Supplementary material for: Paralog-Specific Functions of RPL7A and RPL7B Mediated by Ribosomal Protein or snoRNA Dosage in Saccharomyces cerevisiae
Source: G3 (Bethesda). 2016 Dec 19;7(2):591–606. doi: 10.1534/g3.116.035931 (PMC5295604; doi:10.1534/g3.116.035931)
Supplement: Supplementary file 5 [file 591FileS1.docx]

**Methods for Table S3**

Wild-type and *rpl7a∆::RPL7B rpl7b∆* strains harboring a chromosomal Ty1*his3AI* element were patched onto YPD agar and were incubated at 20˚C for three days to induce retrotransposition. Patches were replicated to SC-HIS agar to select for papillae in which Ty1*HIS3* had transposed. Single colonies were isolated from independent His^+^ papillae, and grown in YPD broth. Genomic DNA was extracted from independent His^+^ colonies (1), and the location of Ty1*HIS3* integration events were identified using thermal asymmetric interlaced (TAIL) PCR according to the method of Liu and Whittier (2), with modifications that have described previously (3). Primer LTR-PROBE (Table S2), which anneals to the U3 region of the Ty1 LTR, was used to sequence TAIL PCR products. Sequencing reads were aligned to the yeast genome using the BLAST tool at the *Saccharomyces* Genome Database ([www.yeastgenome.org](http://www.yeastgenome.org)). The BLAST hits displayed in Table S3 each yielded a significant E-value (<0.05). Prior to TAIL PCR, His^+^ isolates harboring Ty1*HIS3* cDNA within a multimeric Ty1 array were screened via PCR with primers HISOUT3 and TYAOUT2 (3), and were not studied further. Isolates in which Ty1*HIS3* cDNA is present within a multimeric Ty1 cDNA array are noted in Table S3.

**References**

1. **Lõoke M, Kristjuhan K, Kristjuhan A.** 2011. Extraction of genomic DNA from yeasts for PCR-based applications. BioTechniques **50:**325-325.

2. **Liu Y-G, Whittier RF.** 1995. Thermal asymmetric interlaced PCR: automatable amplification and sequencing of insert end fragments from P1 and YAC clones for chromosome walking. Genomics **25:**674-681.

3. **Stamenova R, Maxwell PH, Kenny AE, Curcio MJ.** 2009. Rrm3 protects the Saccharomyces cerevisiae genome from instability at nascent sites of retrotransposition. Genetics **182:**711-723.
